# Female-biased introductions produce higher predicted population size and genetic diversity in simulations of a small, isolated tiger (*Panthera tigris*) population

**DOI:** 10.1038/s41598-023-36849-z

**Published:** 2023-07-11

**Authors:** Eric Ash, Samuel Cushman, Żaneta Kaszta, Erin Landguth, Tim Redford, David W. Macdonald

**Affiliations:** 1 Wildlife Conservation Research Unit, Department of Biology, The Recanati-Kaplan Centre, University of Oxford, Tubney House, Tubney, OX13 5QL Oxon UK; 2 Department of Biological Sciences, Northern Arizona University, 617 S Beaver, Flagstaff, AZ 86011 USA; 3 School of Public and Community Health Sciences, Center for Population Health Research, University of Montana, 32 Campus Drive, Missoula, MT 59812 USA; 4 Freeland Foundation, Lumpini Ville Phahon-Sutthisan, 23/90 7th Floor, Bldg. B, Sutthisan Winitchai Rd., Samsen Nai, Phaya Thai, Bangkok, 10400 Thailand

**Keywords:** Conservation biology, Ecological genetics, Ecological modelling, Population dynamics, Restoration ecology, Ecology, Conservation biology, Ecological genetics, Population dynamics, Restoration ecology

## Abstract

Isolation of wildlife populations represents a key conservation challenge in the twenty-first century. This may necessitate consideration of translocations to ensure population viability. We investigated the potential population and genetic trajectory of a small, isolated tiger (*Panthera tigris*) population in Thailand’s Dong Phayayen-Khao Yai forest complex across a range of scenarios. Using an individual-based, spatially-explicit population modelling approach, we simulate population and genetic trajectories and evaluate the relative impact of translocations from a related population. Population and genetic trajectories in our study were most sensitive to sex and number of individuals translocated and translocation frequency. Translocation of females produced consistently higher population, allelic richness, and heterozygosity compared to equal numbers of males. Despite population increases, declines in allelic richness and heterozygosity across simulations were stark, with simulations predicting a mean decline of allelic richness and heterozygosity of 46.5% and 53.5% without intervention, respectively. Translocations of four females every generation or every other generation were required to prevent substantial heterozygosity declines. While translocations could increase population size, they may fail to prevent long-term loss of genetic diversity in small populations unless applied frequently. This reinforces the importance of incorporating realistic processes of genetic inheritance and gene flow in modelling small populations.

## Introduction

Evolutionary and adaptive processes unfold upon the foundation of genetic diversity^1^, allowing populations to persist in changing landscapes. In order to maintain these processes, effective management of wildlife requires that populations are functionally connected with sufficient numbers to facilitate genetic flow. Failure to do so invariably results in a loss of genetic variation which may undermine population persistence^2,3^.

Effective management of genetic diversity represents a grand and challenging task in the twenty-first century. Populations of many species are suffering both considerable declines as well as isolation caused by habitat loss and fragmentation^4,5^. As a consequence, incidences of inbreeding are likely to increase for many wildlife populations^6^. The potential effects of inbreeding on population persistence is considered to be a long-term challenge. However, a growing body of evidence of acute effects of inbreeding merits greater short-term consideration, particularly for imperiled species in small, isolated populations^7–9^. A lack of genetic diversity has been implicated in phenotypic abnormalities, fertility issues (e.g., cryptorchidism, poor sperm quality), reduced neonatal survival, heart defects, and susceptibility to disease, and may undermine the ability to adapt to changing climate^7,10,11^. The effects of inbreeding may be exacerbated by other issues such as environmental catastrophe^9^ reaching thresholds past which extinction probability increases dramatically^12^.

Such issues may be particularly challenging for wide-ranging and threatened species such as tigers (*Panthera tigris*). Tigers require habitat across sufficiently extensive areas to support large effective populations and the ability to disperse to other areas. This has made tigers susceptible to isolation through large-scale habitat conversion that has occurred over the past century^13–15^. As a result of habitat loss, remaining populations are restricted to relatively few breeding individuals, primarily in protected areas surrounded by a human-dominated matrix^14,15^. While natural gene flow, augmented by the use of habitat corridors, can ameliorate the effects of inbreeding through genetic rescue^9,11^, this may not be possible for many populations without human intervention, such as through translocations.

**Figure 1:**
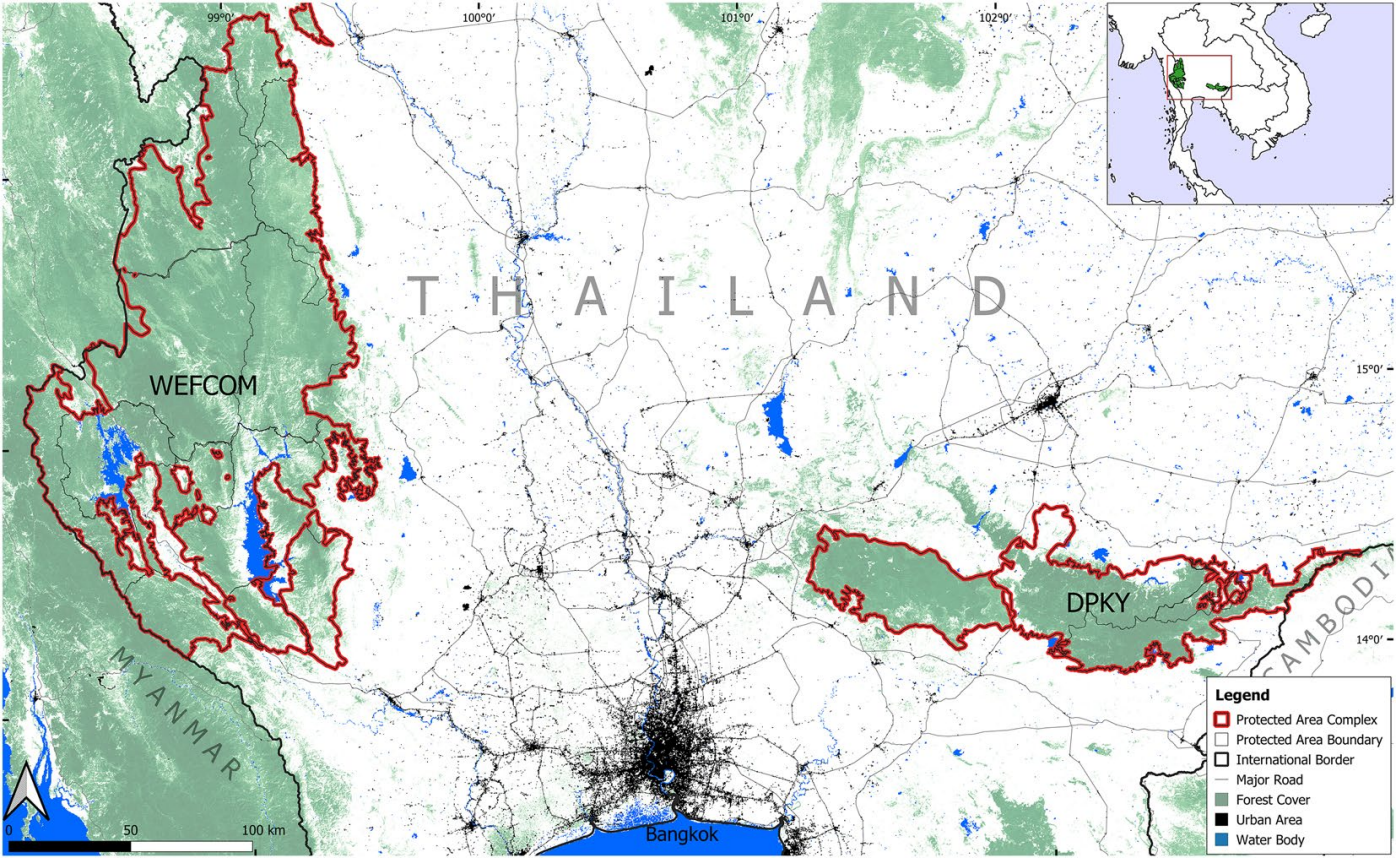
Protected area complexes of regional tiger conservation priority, including the Dong Phayayen-Khao Yai forest complex (DPKY) in eastern Thailand and the Western Forest Complex (WEFCOM). Forest cover (> 50%) from Hansen et al.^64^, urban areas and surface water from SERVIR-Mekong^59^, and major roadways from OpenStreetMap^60^. Map generated in QGIS v3.16.15-Hannover (https://qgis.org).

Practitioners of tiger management and conservation are now faced with a new conservation paradigm in the twenty-first century. In response to irreversible loss of population connectivity, large-scale tiger population management strategies may need to include guidance for investigating the population genetic viability and ascertain whether active management of isolated populations is merited^16–18^. This is particularly important for the Indochinese tiger (*P. tigris corbetti*), one of the most endangered subspecies^15,19^. Once ranging across mainland Southeast Asia, recent estimates suggest the subspecies is potentially limited to < 200 individuals in Myanmar and Thailand^15^. These individuals are largely restricted to two landscapes—the Dawna-Tenasserim landscape along the Thai-Myanmar border (~ 62,000 km^2^, including Thailand’s Western Forest Complex), and the Dong Phayayen-Khao Yai Forest Complex (DPKY, 6155 km^2^) in Eastern Thailand (Fig. 1).

The DPKY landscape was recently found to support a population of 20 (14–33) tigers^20^, likely the last remaining representatives of a metapopulation of *P.t.corbetti* that ranged across eastern Thailand, Cambodia, Lao PDR, and Vietnam^21,22^. The landscape is effectively isolated, with the closest neighboring population—Thailand’s Western Forest Complex (WEFCOM)—approximately 200 km away (Fig. 1). These two landscapes are separated by a well-established human-dominated matrix with few opportunities to re-connect these landscapes via reforestation or other nature-based solutions. DPKY’s low tiger population size and complete isolation merits concern not only over its long-term viability, but also whether current circumstances may precipitate an extinction vortex from which the population would not be able to recover^23,24^.

The situation facing tigers in DPKY may parallel the early stages of one of the most prominent genetic management case studies in felids: the Florida panther (*Puma concolor coryi*). In the early 1990s, the Florida panther had declined to an estimated population of ~ 19–30 individuals^25^, completely isolated from the nearest extant population, now restricted to approximately 5579 km^2^ of habitat^26^. Alarming evidence of inbreeding depression—including kinked tails, dorsal fur whorls, cryptorchidism, and poor sperm quality^7,27^—prompted the translocation of 8 female pumas from Texas^27^. Preliminary evidence suggests this genetic introgression resulted in, at least in the short-term, genetic rescue of the population, which saw population growth and reduced occurrence of abnormalities in admixed individuals^27^.

While translocation of tigers from elsewhere in Thailand to DPKY has been suggested as a potential management strategy to recolonize parts of the landscape, its use as a potential genetic management strategy akin to the Florida panther has not been formally planned. Consideration of such a drastic intervention and potential efficacy would require evaluation of a number of factors, such as baseline genetic variation, sex, number of individuals introduced, translocation frequency, and mortality risk^11,28^. Individual-based population modelling offers an effective means to investigate the influence of translocations or natural migration^27,29,30^. Specifically, models in which movement, mating, dispersal, and genetic inheritance are simulated in a spatially-explicit manner offer a critical advantage. Such models relate these processes to the configuration of real-world landscapes and can enable investigation into the relative effect of various management strategies and landscape change^31–33^.

The DPKY landscape represents a valuable case study for understanding the potential population and genetic trajectory of many tiger populations in which active management to promote gene flow may be required. Immediate threats, such as poaching or loss of habitat, may be of foremost concern in population management strategies^15^. However, threats from continual loss of genetic diversity and associated effects of inbreeding depression could represent a management blind-spot within which extinction vortices may unfold. The case study of the Florida panther and other research suggest understanding and mitigating the effects of population isolation may be more urgent than current management plans suggest^11,18,27^.

In this study, we aim to investigate the potential population and genetic trajectory of a small, isolated breeding population of Indochinese tigers in the DPKY forest complex. Specifically, we utilize an individual-based, spatially-explicit population modelling approach to: (1) quantify the likely population and genetic trajectory of the DPKY tiger population in its current state, and (2) determine the relative impact of translocations of tigers from elsewhere in Thailand on these trajectories. In addition, we evaluate the sensitivity of predictions to: starting genetic variation (APL), genetic relatedness between source and destination populations (SA%), variation in the number and sex of individuals translocated into DPKY (TSEX), frequency of translocations (TFRQ), and mortality risk in translocated individuals (TMOR). To do this, our study explores the theoretical translocation of tigers from Thailand's Western Forest Complex (WEFCOM) landscape, which represents the geographically closest extant population with which DPKY was historically connected. Through this study, we seek to provide a foundation for further conservation and management inquiries relating to genetic management of the tiger population in DPKY and provide insight into considerations for similar spatially-explicit population modelling of other populations.

## Results

**Figure 2:**
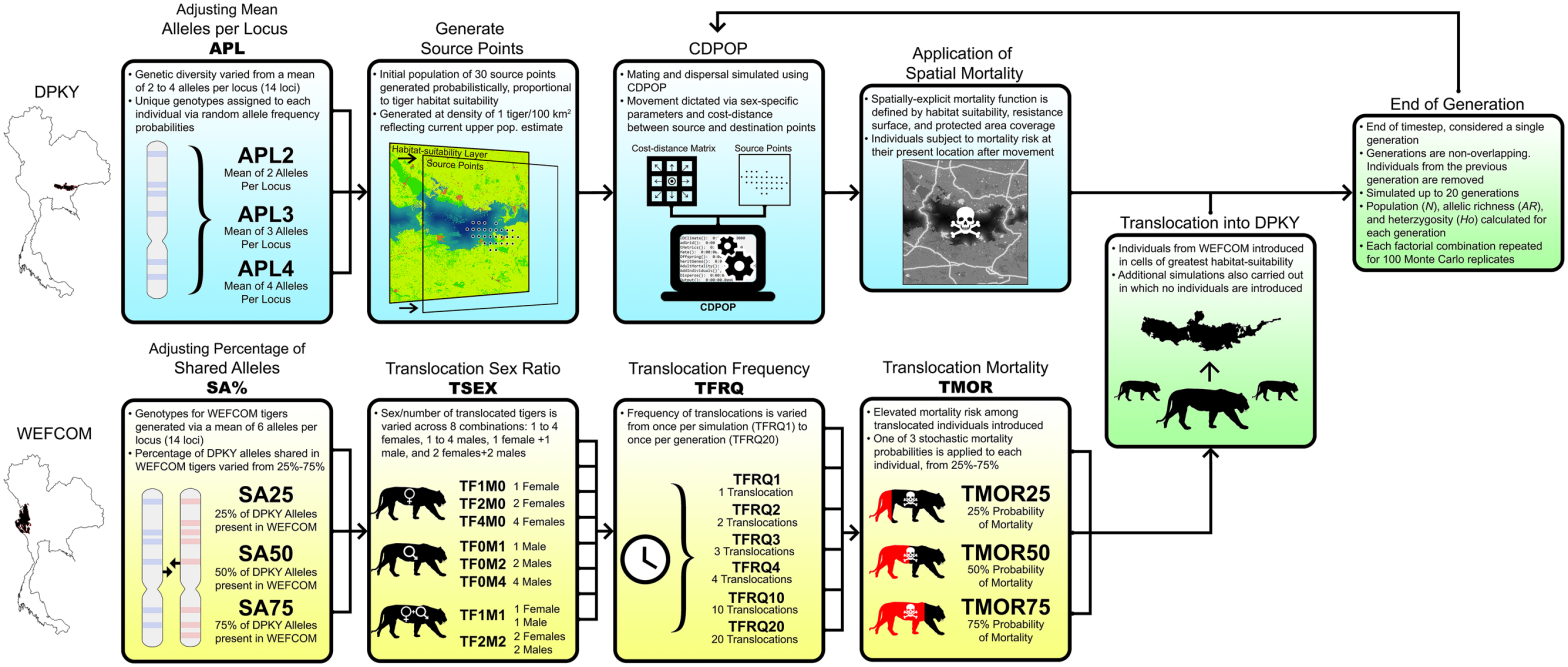
Diagram depicting methods workflow, including development of five factors tested in this study: generation of individual genotypes for in DPKY and adjustment of mean alleles per locus (APL); generation of individual genotypes for translocated individuals from WEFCOM and adjustment of the percentage of shared alleles with DPKY (SA%); adjusting translocation sex ratio (TSEX); adjusting translocation frequency (TFRQ), and adjusting translocation mortality probability (TMOR). Image generated in Adobe Photoshop CC 20.0.0 (https://www.adobe.com)﻿.

Here, we discuss the relative effect of translocations of tigers in the DPKY landscape in terms of: (1) population size and (2) genetic trajectory. For comparison, we evaluate these metrics across levels of each factor, amalgamating all other factors. Definitions and the modelling workflow process are highlighted in Fig. 2.

### Effect of translocations on population trajectory

Simulations in which no translocations occurred (base scenario) produced mean *N* ($\overline{N }$_20_; tiger population) values that increased slightly over 20 generations, from a starting value of 30 individuals and increasing by ~ 14% to 34.32 (Table 1).

**Table 1:** Mean population ($\overline{N }$_*20*_), allele richness ($\overline{AR }$_*20*_), and heterozygosity ($\overline{Ho }$_*20*_) values at generation 20 by factor, with percentage difference relative to scenarios with no translocations﻿.

	$\overline{N }$_*20*_		$\overline{AR }$_*20*_		$\overline{Ho }$_*20*_	
APL						
APL2	38.98		29.36		0.296	
APL3	39.03	+ 0.1%	32.93	+ 12.1%	0.350	+ 18.2%
APL4	38.94	− 0.1%	35.56	+ 21.1%	0.374	+ 26.4%
SA%						
SA0	34.32		21.95		0.250	
SA25	40.09	+ 16.8%	36.51	+ 66.4%	0.370	+ 48.1%
SA50	39.88	+ 16.2%	34.90	+ 59.0%	0.359	+ 43.6%
SA75	40.09	+ 16.8%	33.55	+ 52.9%	0.352	+ 41.0%
TSEX						
TF0M0	34.32		21.95		0.250	
TF0M1	34.48	+ 0.5%	26.87	+ 22.4%	0.294	+ 17.9%
TF0M2	34.15	− 0.5%	29.93	+ 36.4%	0.323	+ 29.4%
TF0M4	34.47	+ 0.4%	33.70	+ 53.5%	0.356	+ 42.7%
TF1M0	39.58	+ 15.3%	30.93	+ 40.9%	0.324	+ 29.8%
TF2M0	43.71	+ 27.4%	37.31	+ 70.0%	0.374	+ 49.9%
TF4M0	50.49	+ 47.1%	45.20	+ 106.0%	0.435	+ 74.1%
TF1M1	39.11	+ 13.9%	34.21	+ 55.9%	0.356	+ 42.5%
TF2M2	44.17	+ 28.7%	41.76	+ 90.3%	0.418	+ 67.6%
TFRQ						
TFRQ0	34.32		21.95		0.250	
TFRQ1	35.35	+ 3.0%	24.58	+ 12.0%	0.275	+ 10.1%
TFRQ2	36.44	+ 6.2%	27.88	+ 27.0%	0.300	+ 20.0%
TFRQ3	37.07	+ 8.0%	30.10	+ 37.2%	0.322	+ 29.0%
TFRQ4	38.33	+ 11.7%	32.76	+ 49.3%	0.343	+ 37.4%
TFRQ10	43.05	+ 25.4%	41.44	+ 88.8%	0.417	+ 67.1%
TFRQ20	49.88	+ 45.3%	53.17	+ 142.3%	0.504	+ 101.8%
TMOR						
TMOR0	34.32		21.95		0.250	
TMOR25	42.51	+ 23.9%	39.41	+ 79.6%	0.397	+ 59.1%
TMOR50	40.30	+ 17.4%	35.55	+ 62.0%	0.365	+ 46.2%
TMOR75	37.24	+ 8.5%	30.01	+ 36.8%	0.318	+ 27.4%

The translocation of new individuals into the population in our simulations produced notably different population trajectories depending on the sex of translocated individuals (TSEX; Table 1). Specifically, population trajectories of simulations in which only males were translocated did not considerably differ from scenarios in which no translocations occurred. Further, increasing the number of males translocated in a given generation did not change this result, with male-only translocations differing in mean *N* at generation 20 ($\overline{N }$_20_) from − 0.5% (TF0M2) to + 0.4% (TF0M4) compared to the base scenario. In contrast, translocations of females produced *N* values that differed considerably from the base scenario with clear differences arising from the number of individuals translocated. In these simulations, $\overline{N }$_20_ was 15.3% (TF1M0) to 47.1% (TF4M0) higher than the base scenario. When equal numbers of males and females were translocated into the population (TF1M1 and TF2M2), the effect on population trajectory was greater than male-only translocations, but less than that of female-only translocations. Specifically, $\overline{N }$_20_ for these scenarios was 13.9% (TF1M1) to 28.7% (TF2M2) higher than the base scenario.

The number of translocations (TFRQ) conducted across simulations also had a noticeable, linear effect on $\overline{N }$_20_ (Table 1), resulting in $\overline{N }$_20_ values 3.0% (TFRQ1) to 28.7% (TFRQ20) higher than the base scenario. Differences in $\overline{N }$_20_ between different translocation mortality thresholds (TMOR) were also relatively linear with $\overline{N }$_20_ ranging from 8.5% (TMOR75) to 23.9% (TMOR25) higher than simulations without translocations.

**Figure 3:**
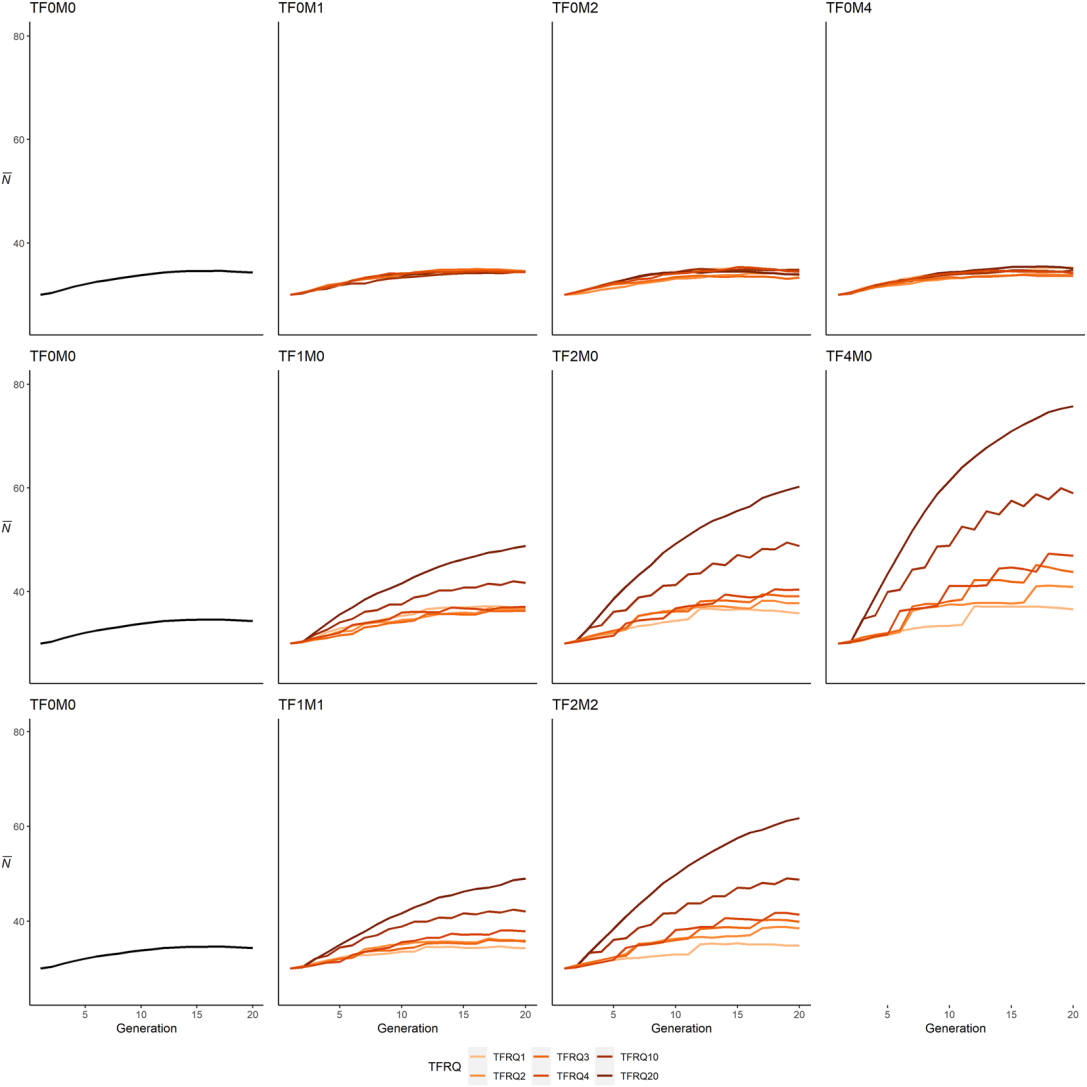
Comparison of mean population ($\overline{N }$) trajectories with no translocations (first column), translocations of males (first row), translocations of females (middle row), and mixed translocations (bottom row), with lines denoting frequency of translocations (TFRQ).

**Figure 4:**
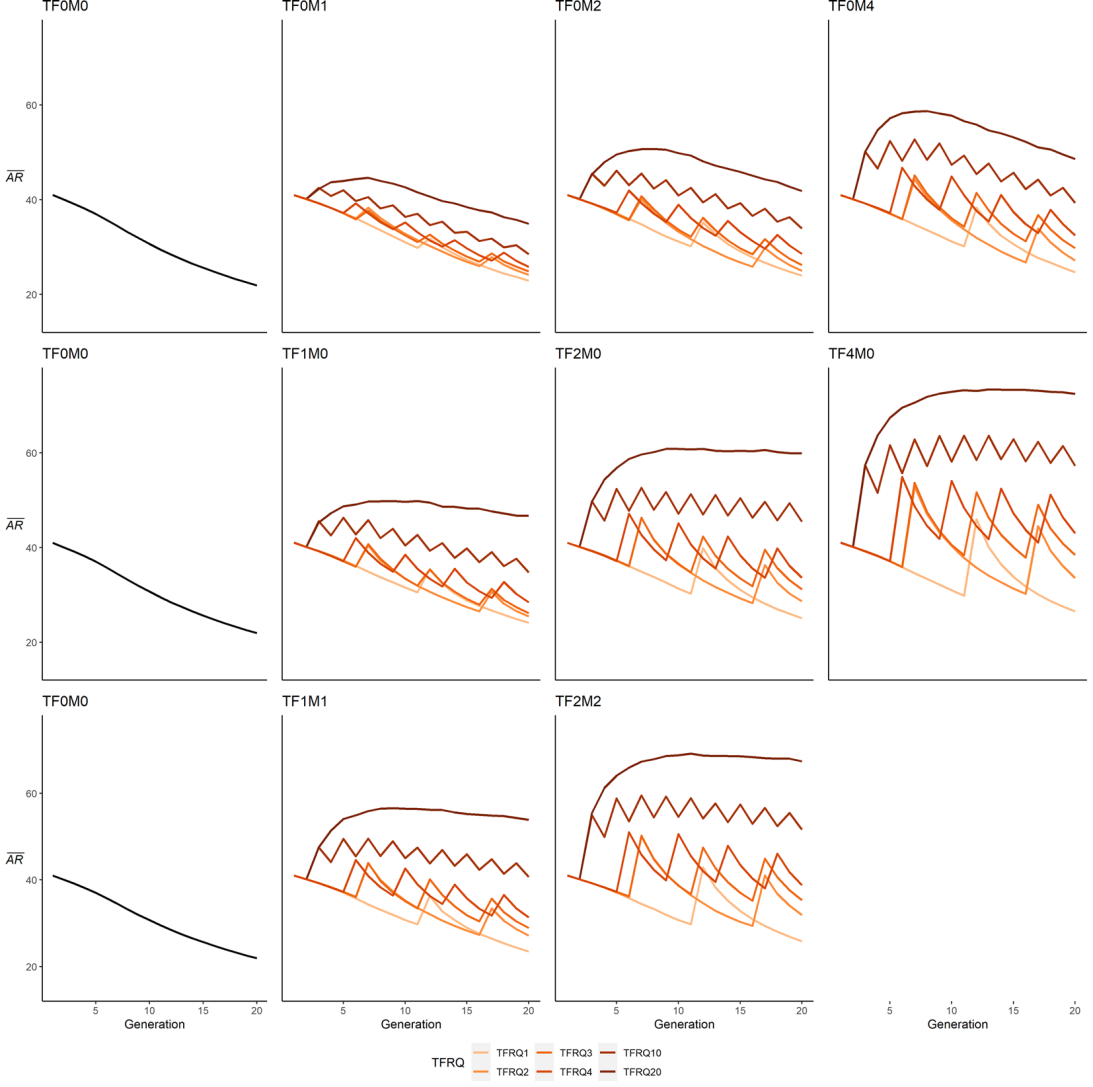
Comparison of mean allele frequency ($\overline{AR }$) trajectories with no translocations (first column), translocations of males (first row), translocations of females (middle row), and mixed translocations (bottom row), with lines denoting frequency of translocations (TFRQ).

The effect of sex and number of individuals translocated into the population (TSEX) are also evident when evaluating $\overline{N }$_20_ in conjunction with the frequency of translocations (TFRQ; Table 2, Fig. 3). In these scenarios, the effect of translocations of relatively large numbers of males on $\overline{N }$_20_ appears non-existent or, at best, marginal (Fig. 4). $\overline{N }$_20_ in these simulations were 2.9% lower (TF0M2-TFRQ3) to 3% higher (TF0M4-TFRQ1) than the base scenario. In contrast, $\overline{N }$_20_ differed dramatically depending on the number of individuals and frequency of translocations of females. In these simulations, $\overline{N }$_20_ was 4.4% (TF2M0-TFRQ1) to 120.7% (TF4M0-TFRQ20) higher than simulations where no translocations occurred.

**Table 2:** Mean population ($\overline{N }$_*20*_), allele richness ($\overline{AR }$_*20*_), and heterozygosity ($\overline{Ho }$_*20*_) values at generation 20 across number/sex of translocated individuals (TSEX) and frequency of translocations (TFRQ), with percentage difference relative to scenarios with no translocations (TF0M0:TFRQ0).

	TSEX	TFRQ	$\overline{N }$_20_		$\overline{AR }$_20_		$\overline{Ho }$_20_	
Male translocations	TF0M0	TFRQ0	34.32	–	21.95	–	0.250	–
	TF0M1	TFRQ1	34.50	+ 0.5%	22.91	+ 4.4%	0.257	+ 3.0%
	TF0M1	TFRQ2	34.45	+ 0.4%	24.15	+ 10.1%	0.269	+ 7.7%
	TF0M1	TFRQ3	34.58	+ 0.8%	24.88	+ 13.4%	0.276	+ 10.6%
	TF0M1	TFRQ4	34.41	+ 0.3%	25.84	+ 17.7%	0.285	+ 14.0%
	TF0M1	TFRQ10	34.44	+ 0.3%	28.51	+ 29.9%	0.310	+ 24.1%
	TF0M1	TFRQ20	34.52	+ 0.6%	34.94	+ 59.2%	0.370	+ 48.1%
	TF0M2	TFRQ1	34.46	+ 0.4%	23.97	+ 9.2%	0.269	+ 7.8%
	TF0M2	TFRQ2	33.79	− 1.6%	24.98	+ 13.8%	0.274	+ 9.7%
	TF0M2	TFRQ3	33.34	− 2.9%	26.22	+ 19.5%	0.289	+ 15.6%
	TF0M2	TFRQ4	34.45	+ 0.4%	28.60	+ 30.3%	0.312	+ 24.8%
	TF0M2	TFRQ10	34.88	+ 1.6%	33.94	+ 54.7%	0.362	+ 44.9%
	TF0M2	TFRQ20	34.00	− 0.9%	41.86	+ 90.8%	0.433	+ 73.6%
	TF0M4	TFRQ1	35.35	+ 3.0%	24.73	+ 12.7%	0.276	+ 10.5%
	TF0M4	TFRQ2	33.80	− 1.5%	27.17	+ 23.8%	0.294	+ 17.8%
	TF0M4	TFRQ3	33.70	− 1.8%	29.81	+ 35.8%	0.322	+ 28.9%
	TF0M4	TFRQ4	34.19	− 0.4%	32.50	+ 48.1%	0.344	+ 37.7%
	TF0M4	TFRQ10	34.71	+ 1.2%	39.34	+ 79.3%	0.409	+ 63.9%
	TF0M4	TFRQ20	35.07	+ 2.2%	48.62	+ 121.5%	0.492	+ 97.2%
Female translocations	TF1M0	TFRQ1	36.93	+ 7.6%	24.12	+ 9.9%	0.271	+ 8.6%
	TF1M0	TFRQ2	36.62	+ 6.7%	25.48	+ 16.1%	0.281	+ 12.5%
	TF1M0	TFRQ3	36.27	+ 5.7%	26.15	+ 19.2%	0.285	+ 14.2%
	TF1M0	TFRQ4	37.12	+ 8.2%	28.40	+ 29.4%	0.303	+ 21.5%
	TF1M0	TFRQ10	41.71	+ 21.5%	34.72	+ 58.2%	0.360	+ 44.0%
	TF1M0	TFRQ20	48.80	+ 42.2%	46.68	+ 112.7%	0.445	+ 78.2%
	TF2M0	TFRQ1	35.84	+ 4.4%	25.11	+ 14.4%	0.279	+ 11.8%
	TF2M0	TFRQ2	37.80	+ 10.1%	28.63	+ 30.5%	0.307	+ 22.9%
	TF2M0	TFRQ3	39.10	+ 13.9%	31.17	+ 42.0%	0.330	+ 32.3%
	TF2M0	TFRQ4	40.44	+ 17.8%	33.63	+ 53.3%	0.348	+ 39.4%
	TF2M0	TFRQ10	48.80	+ 42.2%	45.44	+ 107.1%	0.442	+ 76.9%
	TF2M0	TFRQ20	60.26	+ 75.6%	59.86	+ 172.8%	0.539	+ 115.9%
	TF4M0	TFRQ1	36.57	+ 6.6%	26.51	+ 20.8%	0.294	+ 17.6%
	TF4M0	TFRQ2	40.95	+ 19.3%	33.58	+ 53.0%	0.344	+ 37.6%
	TF4M0	TFRQ3	43.80	+ 27.6%	38.41	+ 75.0%	0.391	+ 56.7%
	TF4M0	TFRQ4	46.86	+ 36.5%	43.02	+ 96.0%	0.423	+ 69.4%
	TF4M0	TFRQ10	58.98	+ 71.9%	57.26	+ 160.9%	0.532	+ 112.9%
	TF4M0	TFRQ20	75.75	+ 120.7%	72.45	+ 230.1%	0.625	+ 150.2%
Mixed translocations	TF1M1	TFRQ1	34.29	− 0.1%	23.48	+ 7.0%	0.264	+ 5.7%
	TF1M1	TFRQ2	35.61	+ 3.8%	27.16	+ 23.7%	0.294	+ 17.9%
	TF1M1	TFRQ3	35.86	+ 4.5%	28.87	+ 31.6%	0.313	+ 25.2%
	TF1M1	TFRQ4	37.86	+ 10.3%	31.32	+ 42.7%	0.332	+ 33.1%
	TF1M1	TFRQ10	42.07	+ 22.6%	40.69	+ 85.4%	0.417	+ 67.1%
	TF1M1	TFRQ20	48.95	+ 42.6%	53.76	+ 145.0%	0.515	+ 106.2%
	TF2M2	TFRQ1	34.87	+ 1.6%	25.83	+ 17.7%	0.289	+ 15.6%
	TF2M2	TFRQ2	38.49	+ 12.2%	31.88	+ 45.3%	0.335	+ 34.1%
	TF2M2	TFRQ3	39.86	+ 16.2%	35.32	+ 60.9%	0.371	+ 48.6%
	TF2M2	TFRQ4	41.36	+ 20.5%	38.73	+ 76.5%	0.398	+ 59.3%
	TF2M2	TFRQ10	48.78	+ 42.1%	51.60	+ 135.1%	0.507	+ 103.1%
	TF2M2	TFRQ20	61.67	+ 79.7%	67.21	+ 206.3%	0.611	+ 144.6%

### Effect of translocations on genetic trajectory

We evaluate the simulated genetic trajectory of the DPKY population in two ways: allelic richness (*AR* or total number of alleles in our population) and observed heterozygosity (*Ho*). In simulations in which no translocations occurred, mean *AR* ($\overline{AR }$) declined by 46.5% from 41 to 21.95 while mean *Ho* ($\overline{Ho }$) declined by 53.5% from 0.538 to 0.250 (Table 1).

The trajectory of genetic diversity differed substantially depending on initial mean number of alleles per locus for DPKY individuals (APL; Table 1). Specifically, scenarios in which an average of two alleles per locus was assigned (APL2) saw a mean *AR* at generation 20 ($\overline{AR }$_20_) 4.9% higher than initial values, while scenarios assigning an average of three (APL3) or four alleles per locus (APL4) had $\overline{AR }$_20_ values 19.0% and 34.6% lower, respectively. Declines in $\overline{Ho }$ among these scenarios were starker, ranging from a decline of 30.2% (APL2), to 40.8% (APL4) by generation 20. Overall, declines in *AR* and *Ho* were more frequent in simulations where DPKY individuals had a higher initial mean number of alleles per locus.

Scenarios in which the percentage of DPKY alleles shared with WEFCOM individuals were varied (SA%) produced drastically different *AR* and *Ho* values compared to the base scenario (Table 1), though these differences were marginal between translocation scenarios. Compared to a decline in $\overline{AR }$ of 46.5% in the base scenario, declines in $\overline{AR }$ ranged from 11.0% (SA25) to 18.2% (SA75) when genetic similarity was varied across translocations. Relative differences in $\overline{Ho }$_20_ values were similar, with base scenario simulations resulting in a decline of 53.6%, while translocation scenarios ranged from a decline of 31.3%, when translocated individuals had fewer shared alleles (SA25), compared to 34.6% with a higher number of shared alleles (SA75).

When evaluating differences in genetic trajectories based on sex and number of translocations (TSEX), similar sex-driven patterns evident in population trajectories also emerge (Table 1). However, in contrast to population, $\overline{AR }$ and $\overline{Ho }$ values arising from the translocation of males differ more substantially from the base scenario. Despite translocations, $\overline{AR }$ and $\overline{Ho }$ declined in most scenarios. In male-only translocation scenarios, $\overline{AR }$_20_ was 17.8% (TF0M4) to 34.5% (TF0M1) lower than initial values. Similarly, $\overline{Ho }$_20_ was lower by 33.88% (TF0M4) to 45.3% (TF0M1) relative to generation 1. Declines were also observed in scenarios involving female-only translocations, though were less substantial. $\overline{AR }$_20_ ranged from 24.6% below (TF1M0) to 10.2% above (TF4M0) starting values while $\overline{Ho }$_20_ remained lower, declining by 19.3% (TF4M0) to 39.8% (TF1M0). In mixed-sex translocation scenarios, $\overline{AR }$_20_ was 16.6% lower than initial values in TF1M1 and 1.8% higher in TF2M2. However, mixed sex translocations produced $\overline{Ho }$_20_ was lower by 22.3% with two females and two males introduced (TF2M2) and lower by 33.9% when halved (TF1M1).

The frequency in which individuals were translocated (TFRQ) also had substantial influence on the genetic trajectory of the population (Table 1). $\overline{AR }$_20_ was 20.1% (TFRQ4) to 40.0% (TFRQ1) lower than initial values when four or fewer translocations were conducted. However, this trend was reversed with translocations every other generation, resulting in $\overline{AR }$_20_ 1.1% higher than initial values, and once-per-generation translocations, which produced an $\overline{AR }$_20_ 29.7% higher than in generation 1. Nonetheless, lower relative $\overline{Ho }$_20_ values were ubiquitous, regardless of the number of translocations, producing declines from 6.4% (TFRQ20) to 48.9% (TFRQ1).

Similar to population trajectories, differences in genetic trajectories with adjustment of mortality probability in translocated individuals (TMOR) were generally linear. For $\overline{AR }$_20_, values were lower by 3.9% (TMOR25) to 26.8% (TMOR75) relative to generation 1, while $\overline{Ho }$_20_ values were lower by 26.2% (TMOR25) to 40.9% (TMOR75; Table 1).

**Figure 5:**
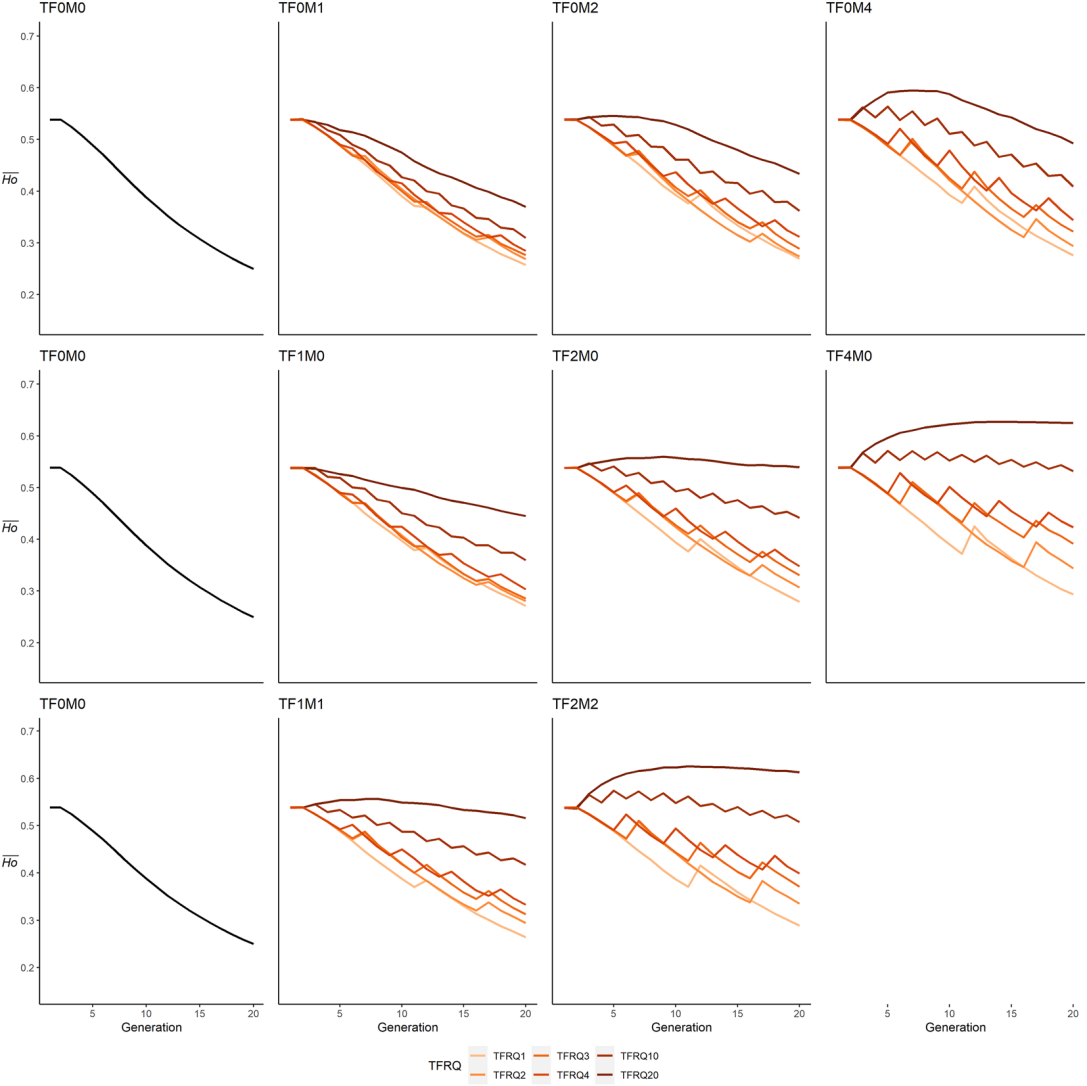
Comparison of mean heterozygosity ($\overline{Ho }$) trajectories with no translocations (first column), translocations of males (first row), translocations of females (middle row), and mixed translocations (bottom row), with lines denoting frequency of translocations (TFRQ).

Evaluating the effect of sex and number of individuals translocated into the population (TSEX), in conjunction with the frequency of translocations (TFRQ), provides additional insight on their effect on genetic trajectories (Figs. 4, 5). Scenarios in which males were translocated one (TFRQ1) to four (TFRQ4) instances across the simulation period resulted in lower $\overline{AR }$ relative to generation 1, with declines of 20.7% (TF0M4-TFRQ4; Table 2) to 44.1% (TF0M1-TFRQ1). Higher relative $\overline{AR }$_20_ were only observed in male-only scenarios in which two (TF0M2; + 2.1%) or four males (TF0M4; + 18.6%) were translocated once per generation (TFRQ20). Conversely, $\overline{Ho }$ declined in male-only translocation scenarios regardless of the number of males or frequency of translocations, lower by 8.5% (TF0M4-TFRQ20) to 52.2% (TF0M1-TFRQ1). In female-only scenarios, $\overline{AR }$_20_ ranged widely from values 41.2% lower than generation 1 (TF1M0-TFRQ1) to 76.7% higher (TF4M0-TFRQ20; Fig. 4). As frequency of translocations increased in these scenarios, $\overline{AR }$_20_ values increased and were higher relative to initial values with once-per-generation translocations of one female (TF1M0-TFRQ20; + 13.8%), two females every other generation (TF2M0–TFRQ10; + 10.8%), and four translocations of four females (TF4M0–TFRQ4; + 4.9%). However, higher $\overline{Ho }$ values were only observed in scenarios in which two (TF2M0; + 0.2%) or four (TF4M0; + 16.1%) females were translocated once per generation (TFRQ20; Fig. 5). For scenarios in which equal numbers of males and females were translocated, $\overline{AR }$_20_ values ranged widely from 42.7% lower than generation 1 (TF1M1-TFRQ1) to 63.9% higher (TF2M2–TFRQ20). In contrast, a higher $\overline{Ho }$_20_ relative to generation 1 only observed in TF2M2-TFRQ20 (+ 13.5%). Additional discussion of results, tables, and figures can be found in Supplementary Materials 2.

## Discussion

Management of small, isolated populations to maintain genetic diversity is emerging as one of the key challenges for the conservation of tigers and other species in the twenty-first century. In this study, we utilize the Dong Phayayen-Khao Yai Forest Complex (DPKY) as a case study in which we used spatially-explicit modelling to simulate tiger population and genetic trajectories. Importantly, our results demonstrate that, while such management interventions can be effective in augmenting tiger population numbers, they may fail to ensure stable or increasing genetic diversity in small populations. This adds further evidence that the one-migrant-per-generation rule of population management may not be sufficient in all biological contexts^34,35^. Notably, substantial differences in the effect of sex on population and genetic trajectories suggest female-biased translocations may be the most effective strategy for augmenting existing populations. Female-biased translocations produced the largest positive population effect and the largest reduction in the rate of decline in genetic diversity. By employing a spatially-explicit, individual-based model that incorporates dispersal, mating, and mortality in relation to a real-world heterogeneous landscape, we were able to simulate the population and genetic consequences of a wide range of realistic introduction scenarios.

Results from our study provide important insight not only into the potential trajectory of the tiger population in DPKY, but also into similar modelling exercises for large felids. First, in the absence of interventions, mean tiger population slightly increased from 30 to ~ 34.32 by the final timestep (generation 20), suggesting the population may have not yet reached carrying capacity under current conditions. However, concurrently, extinction probability in these simulations was relatively high (23%), underscoring the inherent vulnerability of this and other small populations^36,37^ due to demographic stochasticity and paralleling results in Ash et al.^33^.

The effect of translocations on population trajectory varied from negligible, as in the case of several male-only translocation scenarios, to a 120.7% increase in *N* following translocations of four females every generation (TF4M0–TFRQ20). However, these scenarios were mostly insufficient in preventing declines in allelic richness and heterozygosity. This illustrates that population size-focused modelling strategies may be misleading in revealing the degree of risk to small, isolated populations. Specifically, population increases may obscure declines in genetic diversity that may undermine population persistence. This reinforces the importance of incorporating realistic processes of genetic inheritance and gene flow in modelling small populations^27,38^.

The decline in allelic richness and heterozygosity across our simulations was stark. In the absence of tiger translocations, our simulations predicted a mean loss of 46.5% of allelic richness to 21.95% (mean alleles per locus of 1.57) and 53.5% of heterozygosity to 0.250 in 20 generations (~ 100 years). In Thatte et al.^31^, using a similar modelling framework, genetic diversity in tigers across a network of protected areas declined in all scenarios, despite establishment of dispersal corridors.

In comparison with the case study of the Florida panther, mean heterozygosity values in our non-intervention scenarios began to approach levels seen in pre-introgression panthers^7^. Similar to our study, individual-based simulations by van de Kerk et al.^27^ evaluated the degree to which population and genetic trajectories may be affected by the introduction of discrete numbers of female pumas at varying intervals. In their study, introduction of five females every 10 years (~ two generations) resulted in a slight increase in heterozygosity in their study with little additional effect when introducing more females per translocation. In our study, introduction of two to four females every generation was required to elicit a similarly marginal increase in heterozygosity over the same period. However, our study departs from van de Kerk et al.^27^ simulations when comparing non-intervention scenarios. While puma’s heterozygosity declined slightly in van de Kerk et al.^27^, in a 100-year period, declines in our study were much more substantial. This may be due to a number of factors. Notably, these studies model vastly different population sizes, from a starting value of 30 in our study to 133 in van de Kerk et al.^27^. As such, with fewer individuals, these steeper declines are likely driven by more rapid genetic drift in our smaller population^28^. In addition, this disparity may also owe to differences in life-history traits, such as lower litter-size^39^. Importantly, the declines in allelic richness in our simulations occurred despite many scenarios producing varying degrees of population growth, suggesting strategies to maintain genetic diversity through increasing population size alone may be insufficient^18^.

These results demonstrate a key point identified in a study by Chapron et al.^39^ who, in comparing population modelling of pumas and tigers, illustrated that tigers are unlikely to be as resilient to mortality as pumas. While the case study of the Florida panther and evidence of genetic rescue^11,27^ may evoke cautious optimism at potential success for other felid populations, it is likely that similar results in tiger populations may be more difficult to achieve. Modelling of specific populations is required in order to understand the potential impact of these kinds of management strategies. Further, such assessments should be conducted in a spatially-explicit manner, taking into account the specific configuration of the focal landscape and, importantly, how this influences patterns of mortality risk^31–33,40^.

Results of our simulations demonstrated a clear difference in the effect of tiger introductions, with the translocation of females consistently producing higher mean population sizes, allelic richness, and heterozygosity values compared to equal numbers of males. For simulations with low numbers of males or infrequent translocations of males, mean tiger population size was largely indistinguishable from the base scenario, while mitigation of genetic declines were much less effective in comparison to the effects of female translocations. This is likely due to the polygynous breeding behavior of tigers, represented in our simulations, in which the limiting factor in populations is the availability of breeding females^39,41^. This also reinforces the critical importance of breeding females in small populations. Chapron et al.^39^ suggested that mortality rates of resident breeding female tigers exceeding 16% is likely to result in population extinction. This illustrates a quandary in both large-scale management of tiger populations and modelling studies which aim to simulate the potential impact of dispersal corridors to facilitate gene flow between populations. While females may be a limiting factor for populations, corridors linking populations are more likely to be utilized by males, which disperse longer distances^42^. As such, the potential for gene flow across metapopulations may be lower than what may be required to maintain diversity. Further, modelling of population dynamics may only investigate the impacts of males entering a population given this bias in dispersal probability^29^.

The rule of thumb of one-migrant-per-generation required to maintain genetic diversity^34^ has been discussed exhaustively in the literature^28,35,43^. In certain biological systems, the migration of an individual into a population may be beneficial in providing much needed genetic diversity^44^. However, it is possible that this may be an exception rather than a rule and a greater degree of migration may be required. Other evidence suggests that one to ten migrants per generation may be more appropriate^34^ and, in some cases, 20 migrants may be necessary to allay substantial loss of genetic diversity^35^. Our results underscore the complexity of such discussions for tigers. In our study, achieving a higher relative mean allelic richness or heterozygosity was highly dependent not only on the frequency of translocations, but sex as well. Higher mean allelic richness required translocations of females every one to two generations and two or more males every generation. However, higher mean heterozygosity was not observed even in translocations of four males per generation and was only observed when two to four females were translocated in each generation. The specific number of migrants required for a population is likely highly context-specific, even within species and necessitates explicit investigations specific to the population of concern. It is possible that fewer translocations may be necessary for larger populations in which the likelihood of stochastic loss of alleles is comparatively lower.

One of the important limitations of this study is that the current genetic diversity of the population of tigers in DPKY is poorly understood and efforts to undertake genetic analyses have been undermined by low sample size^22^. As such, our simulations are based on a realistic range of mean alleles per locus relative to the closest existing population (WEFCOM). More broadly, as indicated in similar simulations in Kaszta et al.^32^, our modelling framework is best implemented to compare relative differences in scenarios as opposed to predicting population and genetic metrics into the far future with great certainty. The consistent population and genetic trajectories observed in our simulations, despite adjustments for genetic components, suggest this genetic uncertainty has not undermined our objectives.

While genetic diversity in our study declined in most scenarios, this may be an underestimate given that, for simplicity, our study assumes genetic diversity in our source population (WEFCOM) is static. While WEFCOM is large and relatively contiguous at present^45^, a degree of loss of genetic variation would be expected to occur over the time periods simulated in our study^18^. We also did not include the potential effects of inbreeding depression associated with declines in genetic diversity, such as fertility issues observed in other species^7,9,27^ or expression of diploid lethal equivalents^29^. Further, while the effect of inbreeding avoidance^46^ was not incorporated into our models, biases introduced via mate-selection may be an additional factor worth exploring in future assessments.

The substantial declines in genetic diversity of the DPKY tiger population observed in our study is concerning. These declines often far exceeded the 5%-10% reduction in mean heterozygosity over 100–200 years suggested as necessary to avoid inbreeding depression in real-world systems^47,48^. This may be an indication of an inbreeding threat in the DPKY population even in the short-term^6,9^. Felids are known to be particularly sensitive to inbreeding depression^8^ and while evidence exists of the effects of inbreeding depression on captive tigers^49^ the degree to which such issues may manifest in this and other wild tiger populations is poorly understood^22^.

Considering the results of this study, and case studies from other felids, a genetic monitoring plan and assessment is warranted not only to quantify the current genetic state of this population, but also provide a foundation for the development of clear management and recovery strategies^22^. This study presents an exercise to investigate the relative effect of various potential management scenarios on the DPKY tiger population. In reality, such interventions are controversial, costly, logistically challenging, and would require a population that meets genetic rescue guidelines prior to any intervention^11^. A key constraint to possible implementation of the measures we explored in this work is the limited opportunities to source tigers for translocations given low extant population sizes. Any initiative that seeks to capture and translocate tigers across large distances must ensure that such efforts would not undermine the conservation of tigers in the landscape from which they are removed^50^. This is of particular relevance as, while WEFCOM remains the closest extant tiger population to DPKY, its current estimated population of 125–149 adults^15^ is vulnerable. Efforts to source tigers for translocation would require meticulous planning, comprehensive engagement with a number of key stakeholders, and the most up-to-date data from which decisions could be made^50^. Further, factors such as cost, animal behavior, age class of introduced individuals, monitoring, availability of prey, and other factors would warrant careful consideration. Insight from this study may prove a useful starting point from which such discussions may emerge.

Our results identify serious concerns regarding the long-term genetic trajectory for the Indochinese tiger subspecies. Currently, the subspecies numbers approximately 145–177 individuals in Thailand and > 22 in Myanmar^15^. In addition, there is no coordinated captive breeding program for the subspecies nor substantial representation in captivity^51^. Thus, the subspecies lacks reservoirs of genetic variation in captivity, unlike other species, e.g. the Amur tiger^52^. Evidence suggests that existing subpopulations may not meet minimum viable population size thresholds^38^. Even for one of the largest single populations of tigers globally (Western-Ghats, India^18^), one study suggests an unrealistic amount of population growth and size would be required to prevent loss in genetic diversity.

A controversial option, akin to initial discussions of genetic rescue of the Florida panther^53^, would be introgression from individuals from other subspecies into populations in Southeast Asia. Whether potential benefits would outweigh potential risks, such as outbreeding depression, and whether prevailing taxonomic designations should guide genetic management^15^, would necessitate rigorous debate and additional research. High-level coordination of active management across populations may represent a key component of tiger conservation in the future. Ultimately, however, even if tiger population managers and conservation practitioners address these existential questions, such debates will matter little if the threats that have driven many tiger populations to the brink are not effectively addressed.

## Methods

### Overview

The primary objective of our analysis is to investigate the potential population and genetic trajectory of a small, isolated tiger population and relative effect of translocations of individuals from a related population. Using an individual-based, spatially-explicit population modelling approach, we evaluate the relative impact of translocations via five factors: (1) Starting average alleles per locus for the DPKY population (APL); (2) Percentage of DPKY alleles shared with translocated individuals (SA%), (3) Number/sex of translocated individuals (TSEX); (4) Frequency of translocations (TFRQ); and (5) Mortality probability of translocated individuals (TMOR). In addition, to understand potential population and genetic trajectories without translocations, we carried out simulations in which no translocations occurred. Our modelling workflow is visualized and described in Fig. 2.

The focal landscape for our study is the Dong Phayayen-Khao Yai Forest Complex (DPKY), located in Eastern Thailand (Fig. 1). The landscape consists of five protected areas across 6155 km^2^—Khao Yai National Park, Thap Lan National Park, Pang Sida National Park, Ta Phraya National Park and Dong Yai Wildlife Sanctuary—and currently supports a breeding population of Indochinese tigers (*P. t. corbetti*)^54^.

Given a lack of suitable representation of the subspecies in captivity^51^ and lower relative success of introducing large carnivores from captivity into the wild^55^, we simulated theoretical translocations from in situ stock. Our study explores the theoretical translocation of tigers from Thailand’s Western Forest Complex (WEFCOM; 19,666 km^2^), currently the closest tiger population to DPKY. WEFCOM is home to the largest remaining population of tigers in mainland Southeast Asia, currently supporting an estimated 125–149 adults^15^. Consisting of 17 contiguous protected areas (Fig. 1), WEFCOM is situated within the Dawna-Tenasserim Landscape (DTL), potentially linking it to other protected areas in both Thailand and Myanmar. Haplotype COR1 shared between DPKY, western Thailand, and specimens from Cambodia and Vietnam, suggests a connected historical population of the subspecies across the region^21,22^. However, observed genetic structure implies that vast habitat conversion, particularly over the past half century, has effectively separated DPKY from WEFCOM.

### Adjusting mean alleles per locus (APL)

While efforts have been made to collect and sequence genetic samples from DPKY, detailed information regarding the genetic diversity of DPKY is not available^22^. In light of this uncertainty, we varied genetic diversity in our study population across a range of plausible values. Given its isolation and smaller population size, we assumed a lower genetic diversity compared to the larger and relatively contiguous WEFCOM population (average of 6 alleles per locus across 14 Loci and observed heterozygosity, *Ho*, of 0.562)^22^. Thus, for DPKY, we varied expected mean alleles per locus (APL) across three levels: two alleles per locus (APL2), three alleles per locus (APL3) and four alleles per locus (APL4). For each scenario, the number of alleles for each locus was generated via a Poisson probability draw with a mean corresponding to either two (APL2), three (APL3), or four (APL4) mean alleles per locus. Allele frequency probabilities were determined by a generating random number between 0 and 1 for each allele, and by dividing the result by the sum of all random numbers generated at that locus. The final allele frequency probability value was then used by the *intgenesans* function in the program CDPOP (described below) to generate unique genotypes for each individual in the simulation.

### Generate source points

The distribution of an initial 30 source points, reflecting upper estimates of the current tiger population^20^, was generated probabilistically and proportional to predicted tiger habitat suitability^56^. Density of individuals in our simulations was constant with occupiable grid cells distributed at a resolution of 10 km^2^ or maximum density of 1 tiger per 100 km^2^.

### Initialize simulations—CDPOP

Simulations were carried out in CDPOP (Cost Distance POPulations), an individual-based, spatially-explicit framework which models mating, dispersal, and mortality across a defined number of timesteps^57^. In each timestep, defined in our study as a single generation, CDPOP simulates movement of individuals as a function of sex-specific movement parameters for mating and dispersal, constrained by the cost-distance between source and destination points via a resistance surface. The resistance surface used for this study was developed and utilized in Ash et al.^56,58^, defining step-wise cost to movement whereby high pixel values impart high resistance to movement and low values little resistance. The resistance surface was developed at 250 m resolution and was based on the 2018 SERVIR–Mekong Regional Land Cover Monitoring System (RLCMS^59^). Land cover was reclassified into dense forest (resistance value of 1), scrub forest (resistance of 20), agriculture/village matrix (resistance of 50), reservoirs/surface water (resistance of 80), and urban areas (resistance of 100). Minor (resistance of 30) and major roads (resistance of 100^60^) were also included. Cost-distance matrices, defining cost to movement between all points, were generated from this resistance surface in the program UNICOR (Universal Corridor Network Simulator)^61^. The number of offspring following breeding was determined via a normal distribution draw with mean of three and standard deviation of two. These and other parameters were borrowed from other published studies on tigers and related genera^31,32,40^.

### Application of spatial mortality

Following individual movement and breeding in each timestep, we applied a spatially-explicit mortality function defined by local, empirically-based predicted tiger habitat suitability (MSSO model from Ash et al.^56^), landscape change scenario, resistance surface, and protected area coverage. This function (MH10) emerged as the most plausible function among those assessed in Ash et al.^33^, a study which underscored the importance of incorporating spatial mortality-risk in individual-based population modelling studies.

### End of generation

Each timestep in our model is considered an individual generation. Generations are non-overlapping and all remaining adults from the previous generation were removed from simulations at each timestep. Simulations were repeated for up to 20 generations.

### Translocations—adjusting percentage of shared alleles (SA%)

Following the same genotyping process above, we generated genotypes for hypothetical translocated individuals from WEFCOM with 14 loci and an average of 6 alleles per locus, corresponding to values from Klinsawat^22^. Due to the lack of comprehensive genetic sampling between these populations, particularly for DPKY, their degree of genetic relatedness at identical loci is unknown. Thus, when generating alleles for each locus between populations, we varied the percentage of DPKY alleles present in simulated WEFCOM individuals (SA%) at three levels: 25% (SA25), 50% (SA50), and 75% (SA75). It is expected that WEFCOM and DPKY populations would share a substantial portion of alleles given they were historically connected prior to twentieth century population declines and habitat loss.

### Translocations—translocation sex ratio (TSEX)

In order to explore the impact of the number and sex of individuals translocated into the population (TSEX), we tested eight translocation scenarios in which the following number of individuals were translocated: one male (TF0M1), two males (TF0M2), four males (TF0M4), one female (TF1M0), two females (TF2M0), four females (TF4M0), one male with one female (TF1M1), and two males with two females (TF2M2).

### Translocations—translocation frequency (TFRQ)

We also tested various frequencies of translocations (TFRQ), with theoretical translocations occurring once per simulation (TFRQ1; generation 10), twice (TFRQ2; generations 5 and 15), three times (TFRQ3; at generations 5, 10, and 15), four times (TFRQ4; generations 4, 8, 12, and 16), 10 times (TFRQ10; every other generation), and 20 times (TFRQ2; once per generation).

### Translocations—translocation mortality (TMOR)

Given that introduction of animals into a new environment may induce an elevated risk of mortality^62^, we applied one of three stochastic mortality probabilities for each translocated individual: 25% (TMOR25), 50% (TMOR50), and 75% (TMOR75). In R^63^, each translocated individual in CDPOP input files was assigned a randomly generated number between 0 and 1. If this randomly generated number fell below a given threshold (i.e., < 0.25 for TMOR25, < 0.5 for TMOR50, and < 0.75 for TMOR75) the individual was removed. This stochastic mortality probability was applied in all cases when one or more individuals are introduced (TSEX—TF0M1, TF0M2, TF0M4, TF1M0, TF2M0, TF4M0, TF1M1, TF2M2) and at all translocation frequencies (TFRQ—TFRQ1, TFRQ2, TFRQ3, TFRQ4, TFRQ10, TFRQ20).

### Translocation into DPKY

Locations for translocations were determined by prioritizing points located in cells of greatest predicted habitat suitability based on a scale- and shape-optimized model^56^. If a given point was occupied at the time of translocation, placement of the translocated individual was random among available grid cells in the landscape. If a population reached carrying capacity during a generation in which a translocation was to occur, individuals would not be translocated into the DPKY population for that generation.

### End of simulations

We conducted 28,800 simulations of 20 generations in which no translocations occurred. Further, each combination of factors—APL, SA%, TSEX, TFRQ, and TMOR—was simulated over 20 non-overlapping generations and repeated over 100 Monte Carlo replicates (129,600 simulations of 20 generations). For each simulation, we calculated and compared differences in population size (*N*), total number of alleles in the population or allelic richness (*AR*), and observed heterozygosity (*Ho*). A full list of parameters can be found in Supplementary Materials 1. All observations were made via simulation results and no animals were directly involved in this study.

## Supplementary Information

Supplementary Information.

## Data Availability

The datasets used and/or analysed during the current study available from the corresponding author on reasonable request.
