# Supplementary material for: Female-biased introductions produce higher predicted population size and genetic diversity in simulations of a small, isolated tiger (Panthera tigris) population
Source: Sci Rep. 2023 Jul 11;13:11199. doi: 10.1038/s41598-023-36849-z (PMC10336066; doi:10.1038/s41598-023-36849-z)
Supplement: Supplementary file 1 — Supplementary Information. [file 41598_2023_36849_MOESM1_ESM.pdf]

Supplementary Materials 1 – Model Overview

In this section, we provide a description of our individual-based model and approach via the OOD (Overview, Design concepts, Detail) protocol, as described by Grimm et al. [1,2].

1. Purpose

The purpose of our modelling approach is to (1) quantify the likely population and genetic trajectory of the tiger population of the Dong Phayayen-Khao Yai Forest Complex (DPKY) in its current state and (2) to determine the relative impact of translocations of tigers from elsewhere in Thailand on these trajectories. In addition, we aimed to evaluate the sensitivity of predictions to starting genetic variation (APL), genetic relatedness between source and destination populations (SA%), variation in the number and sex of individuals translocated into DPKY (TSEX), frequency of translocations (TFRQ), and mortality risk in translocated individuals (TMOR).

2. Entities, state variables, and scales

Our models utilize the program CDPOP (Cost Distance POPulations [3]) which uses an individual-based, spatially-explicit framework to model mating, genetic inheritance, dispersal, and mortality through space and time. Individuals are characterized by several state variables (see Table 1), including: a unique alphanumeric identifier, sex (M/F), location (coordinates), genotype data for user specified number of loci and alleles, and territory unit. Females mate with a single male and males can mate with multiple females in a given timestep. The number of offspring is drawn probabilistically from a normal distribution (mean of 3 and standard deviation of 2, with a 1:1 sex-ratio (Thatte et al. [4] ; Tables 1 and 2). Generations are non-overlapping and, therefore, we do not distinguish individuals by age class.

All individuals in our model are assigned unique genotypes based on 14 theoretical loci with an average number of alleles per locus determined by one of several potential values. The number of alleles for each locus was generated via a Poisson probability draw with a mean corresponding to either two (APL2), three (APL3), or four (APL4) mean alleles per locus. Allele frequency probabilities were determined by a generating random number between 0 and 1 for each allele, and by dividing the result by the sum of all random numbers generated at that locus. The final allele frequency probability value was then used by CDPOP to generate unique genotypes for each individual in the simulation (*intgenesans* function in CDPOP). The same process was repeated to simulate genotypes for translocated individuals from WEFCOM with 14 loci and an average of 6 alleles per locus, corresponding to values from Klinsawat [5]. Due to the unknown degree of genetic relatedness between DPKY and WEFCOM populations at identical loci, when generating alleles for each locus between populations, we varied the percentage of DPKY alleles present in simulated WEFCOM individuals (SA%) at three levels: 25% (SA25), 50% (SA50), and 75% (SA75).

Occupiable locations in our study extent were determined by resampling a cost-distance kernel of 45,000 cost-distance units, originating from DPKY's contiguous forest boundary, to a resolution of 10km. These territorial units were then converted to points representing potential tiger home range centroids at a theoretical population density of 1/100 km^2. For simplicity, maximum carrying capacity density is assumed to be constant over time.

Spatial patterns of the study landscape, with which individuals in simulations would interact, were defined in a resistance surface. A resistance surface reflects step-wise cost to individual movement, with high pixel values representing high resistance to movement and low values imparting little resistance. In our study, resistance surfaces were developed at a 250m resolution. We utilized a base resistance surface developed by Ash et al. [6] and utilized in Ash et al. [7] as the most representative of likely patterns of resistance among

four candidate models. This expert-based surface was based on 2018 SERVIR–Mekong Regional Land Cover Monitoring System (RLCMS [8]) land cover data, classified into dense forest (resistance value of 1), scrub forest (resistance of 20), agriculture/village matrix (resistance of 50), reservoirs/surface water (resistance of 80), and urban areas (resistance of 100). Minor (resistance of 30) and major roads (resistance of 100; [9]) were also included.

Individual movement is defined by sex-dependent movement parameters, differentiated for mating and dispersal behaviour. These and other simulation parameters in our models were based on other studies on tigers and other wide-ranging felids [4,10,11]. The CDPOP model simulates mating among individuals via probabilistic selection of mating pairs based on the cost distance between their locations across the resistance surface. Individuals that are in close proximity in cost distance are more likely to mate than those that are farther, with the probability defined by the mating distance function and maximum distance (see Table 1). Dispersal of offspring produced in mating is also probabilistic based on cost distance, with locations in close cost-proximity more likely to be selected than those that are far, governed by the specified dispersal function and maximum cost threshold.

Movement to find mates was simulated as an inverse square of the cost distance with a threshold adjusted by multiplying 12000m (as per Thatte et al. [4]) by the mean resistance per unit area. Movement of dispersing individuals in our model is sex-specific and uses a negative exponential function with two maximum dispersal distances of 200,000 cost units for females and 600,000 cost units for males. Simulated movement across the landscape in this approach was limited by landscape resistance, defined by individual movement and the cost-distance between occupiable locations. For this purpose, a cost-distance matrix was generated from the resistance surface (250m resolution) and all occupiable points (defined above based on a maximum density of 1 individual per 100km^2).

Individuals in our models are included in our simulations in two ways. First, an initial DPKY population (timestep 1) was generated via a sample of 30 points, representing the upper range of the current tiger population estimates within DPKY boundaries (Ash et al. [12] described further in initialization). Second, in scenarios simulating translocations, individuals are added to the population via the *intgenesans* function in CDPOP. These individuals are assigned genotypes based on allele frequencies defined above. The number and sex of individuals was defined by one of several translocation scenarios (TSEX) being tested – one male (TF0M1), two males (TF0M2), four males (TF0M4), one female (TF1M0), two females (TF2M0), four females (TF4M0), one male/one female (TF1M1), and two males/two females (TF2M2). Individuals were introduced at one of six frequencies, occurring once per simulation (TFRQ1; generation 10), twice (TFRQ2; generations 5 and 15), three times (TFRQ3; at generations 5, 10, and 15), four times (TFRQ4; generations 4, 8, 12, and 16), 10 times (TFRQ10; every other generation), and 20 times (TFRQ2; once per generation). To evaluate potential effects of elevated mortality risk to translocated individuals, one of three stochastic mortality probabilities was applied to each translocated individual: 25% (TMOR25), 50% (TMOR50), and 75% (TMOR75). In R [13], each translocated individual in CDPOP input files was assigned a randomly generated number between 0 and 1. If this randomly generated number fell below a given threshold (i.e., <0.25 for TMOR25, <0.5 for TMOR50, and <0.75 for TMOR75) the individual was removed.

One time step in our simulation represents one non-overlapping generation within the population and simulations were run for 20 generations (approximately 100 years, as per Thatte et al. [4]).

3. Process overview and scheduling

Our simulations were carried out in CDPOP in single, distinct time-steps, representing a non-overlapping generation. The CDPOP simulation works in several discrete steps within each simulated generation:

1. Mating pairs are selected probabilistically according to the mating function described above.
2. These mating events produce offspring based on the demographic function described above, and with Mendelian recombination of genotypes from their parents.
3. The adults die, as this is a non-overlapping generation model.
4. The offspring disperse and occupy adult home range locations governed by the dispersal functions described above.
5. These individuals are then subjected to a mortality filter based on the mortality function. Mortality of individuals would be determined by one of two processes, probability of mortality at a given location or the absence of available territories. For the former, this was dependent on a given mortality function which defines the probability of mortality spatially via certain key factors (See Supplementary Materials 2, Ash et al. [7]). If all territories in a simulation are occupied at the end of the time-step, any remaining individuals would be removed.

Further, in simulations in which translocations occur, further steps are conducted:

6. Introduced individuals are assigned unique genotypes via allele frequency files.
7. Individuals are assigned locations in the landscape based on cells of greatest habitat suitability [14]. If a given point is occupied at the time of translocation, placement of the translocated individual was random among available grid cells in the landscape. If a population reached carrying capacity during a generation in which a translocation was to occur, individuals would not be introduced into the DPKY population for that generation.

At the end of these steps a new set of adults is distributed in the landscape and these steps are then iterated across 20 generations. At each generation we track the number of individuals in the population (*N*), their locations, allelic richness (*AR*), and observed heterozygosity (*Ho*). The entire process was repeated for each factor combination (APL, SA%, TSEX, TFRQ, and TMOR) across 100 Monte Carlo replicates.

4. Design concepts

Basic Principles: Demographic patterns governing species population dynamics and genetic structure are dependent on spatially-dynamic processes across heterogeneous landscapes. Spatially-explicit models can enable understanding of the effect of translocation of individuals into a population on population and genetic structure in relation to the specific spatial configuration of a real-world landscape.

Emergence: The trajectory of populations through simulations is governed by rules pertaining to individual behaviour, mating, dispersal, mortality risk, and location in the landscape which the individual occupies. Importantly, however, predictions may be sensitive to variations in key factors tested in our models - specifically, starting average alleles per locus for individuals in DPKY (APL), and percentage of DPKY alleles shared with WEFCOM (SA%), number/sex of individuals (TSEX), frequency of translocations (TFRQ), and mortality of translocated individuals (TMOR) - and their interactions.

Sensing: Dynamics in CDPOP are probabilistic functions of location and proximity. Individuals respond based on specified probabilistic functions governing mating distances, dispersal distances and mortality risk across the landscape. The modelling includes feedback between individuals, specifically in the selection of mating pairs based on proximity in cost distance, sex, and reproductive state.

Interaction: Interactions between individuals are of two kinds. First, mating occurs between individuals of opposite sexes that are selected based on cost proximity and reproductive state (e.g., adults and females that have not already mated in that time step). Second, dispersal is limited to territories not already occupied, limiting population density and leading to density-dependent mortality if no available territories are accessible within the dispersal capability of the individual.

Stochasticity: Individual life-history and behavioural characteristics emerge stochastically in simulations, governed by probabilistic draws from offspring, sex, movement, and mortality parameters.

Observation: The primary metrics of interest were simulated population values (*N*), allelic richness (*AR*), and observed heterozygosity (*Ho*) at each timestep (generations 1-20). These values were compared across each of the factors tested (APL, SA%, TSEX, TFRQ, and TMOR) and their interactions.

5. Initialization

An initial population (timestep 1) was generated via a sample of 30 points, representing the upper range of the current tiger population estimates within DPKY boundaries [12]. This was generated probabilistically and proportional to predicted tiger habitat suitability. Source point locations were determined by subtracting a random raster, with values from 0-1, from a rescaled multi-scale- and shape-optimized tiger habitat suitability model developed by Ash et al. [14] for DPKY, with values reflecting predicted presence on a scale from 0 to 1. We then selected a random sample of 30 occupiable points (15 males and 15 females) from resulting pixels with values greater than 0 (e.g., Macdonald et al. [15]; Kaszta et al. [11]).

6. Input data

The model does not use input data to represent time-varying processes.

7.Submodels

One component of the study is to evaluate the sensitivity of predictions of tiger population size and genetic variation across key factors. These include varying starting average alleles per locus for individuals in DPKY (APL; three levels), percentage of DPKY alleles shared with WEFCOM (SA%; four levels), the number/sex of individuals (TSEX; nine levels), frequency of translocations (TFRQ; seven levels), and mortality of translocated individuals (TMOR; four levels). We provide greater detail on the method and extent of variation in these factors in both the main text and in Online Resource 2.

Table 1 Parameters used in CDPOP setup file for simulations

	Setup File	
Parameter Name	***Parameter Value***	***Comment***
xyfilename	SOURCEPOINTFILE	This is a reference to the name of the source points xy file. In scenarios in which translocations are tested, an xy file is provided for each generation, starting with an initialization xy file and including xy files for each set of individuals translocated into the population.
agefilename	AGEVARSFILE	See parameters for agevars file below.
mcruns	10	We incorporate 10 source points to account for stochasticity and unknowns. Each set of source points are run ten times, producing 100 MC simulations.
looptime	20	Number of generations to be simulated
output_years	1	
gridformat	cdpop	
cdclimgentime	0|1|2|3|4|5|6|7|8|9|10|11|12|13|14|15|16|17|18|19|20	
matecdmat	CDMATRIXFILE	CDMatrix file generated for each pair of available points, resistance surface, and resolution
dispcdmat	CDMATRIXFILE	As Above
matemoveno	2	2 = Inverse Square (1 / (Cost Distance^2)). This function gets rescaled to the min and threshold of the inverse square cost distance.
matemoveparA	1	
matemoveparB	1	
matemoveparC	1	
matemovethresh	12000 x Mean Resistance	Mate movement threshold adjusted by multiplying 12000m (as per Thatte et al. [4] by mean resistance per unit area.
output_matedistance	N	
sexans	Y	
Freplace	N	
Mreplace	Y	
philopatry	N	
multiple_paternity	N	
selfans	N	
Fdispmoveno	5	5 = Negative Exponential (parA * 10^(-parB * Cost Distance)). This function gets rescaled to the min and threshold of the negative exponential cost distance.
FdispmoveparA	0.4	
FdispmoveparB	1.59E-06	
FdispmoveparC	1	
Fdispmovethresh	2000000	
Mdispmoveno	5	5 = Negative Exponential (parA * 10^(-parB * Cost Distance)). This function gets rescaled to the min and threshold of the negative exponential cost distance.
MdispmoveparA	0.4	
MdispmoveparB	5.62E-07	
MdispmoveparC	1	
Mdispmovethresh	6000000	

Parameter Name	***Parameter Value***	***Comment***
offno	5	Normal distribution draw with mean and sigma for fecundity given in the Agevars file (See below).
Femalepercent	50	Percent number of females born in each litter. This is a random assignment from given percentage.
EqualsexratioBirth	N	
TwinningPercent	0	
popModel	exp	'exp' – or exponential growth where n(t+1) = birth-rate * n(t) – death-rate * n(t). Population numbers can reach the set carrying capacity of the number of individuals in the XY file, but not exceed this number.
R	1	
K_env	5000	
subpopmortperc	0|0|0|0	
muterate	0.0001	Mutation rate used from Thatte et al. [4]).
mutationtype	forwardbackward	Used from Thatte et al. [4].
loci	16	14 loci plus two dummy loci for incorporating spatial mortality risk
intgenesans	file OR file_introduce	*File* is used for scenarios in which no translocations occur. For scenarios simulating translocations, *file_introduce*
allefreqfilename	ALLELEFREQUENCYFILE	This is a reference to the name of the allele frequency file used to generate unique genotypes for each individual in the population. In scenarios in which translocations are tested, an allele frequency file is provided for each generation, starting with an initialization allele frequency file and including allele frequency files to generate genotypes for an individual translocated into the population.
alleles	-	Number of alleles per locus. Dependent on scenario tested in which alleles are generated via a Poisson probability draw. Generated with a mean corresponding to either two (APL2), three (APL3), or four (APL4) mean alleles per locus for DPKY and six alleles per locus for WEFCOM (translocated individuals).
Mtdna	N	
startGenes	0	
Cdevolveans	1	Used in conjunction with dummy loci to simulate heterogeneity in spatial mortality risk across the landscape.
startSelection	0	
betaFile_selection	N	
Epigeneans	N	
startEpigene	0	
betaFile_epigene	N	
Cdinfect	N	
Transmissionprob	0.5	

Table 2 Agevars parameters file for CDPOP simulation setup

AgeVars			
Age class	0	1	
Distribution	0	1	
Male Mortality	0	100	
Female Mortality	0	100	
Mean Fecundity	0	3	Mean young per female – Used from Thatte et al. [4].
Std Fecundity	0	2	Standard deviation of young per female – Used from Thatte et al. [44].
Male Maturation	0	1	
Female Maturation	0	1	

References

1. Grimm, V. *et al.* A standard protocol for describing individual-based and agent-based models. *Ecol. Modell.* **198**, 115–126 (2006).

2. Grimm, V. *et al.* The ODD protocol: A review and first update. *Ecol. Modell.* **221**, 2760–2768 (2010).

3. Landguth, L. E. & Cushman, S. A. cdpop: A spatially explicit cost distance population genetics program. *Mol. Ecol. Resour.* **10**, 156–161 (2010).

4. Thatte, P., Joshi, A., Vaidyanathan, S., Landguth, E. & Ramakrishnan, U. Maintaining tiger connectivity and minimizing extinction into the next century: Insights from landscape genetics and spatially-explicit simulations. *Biol. Conserv.* **218**, 181–191 (2018).

5. Klinsawat, W. Phylogeography and landscape genetics of tigers (Panthera tigris) and Asian elephants (Elephas maximus) in Thailand. (University of Minnesota. PhD Thesis., 2016).

6. Ash, E., Cushman, S. A., Macdonald, D. W., Redford, T. & Kaszta, Ż. How Important Are Resistance, Dispersal Ability, Population Density and Mortality in Temporally Dynamic Simulations of Population Connectivity? A Case Study of Tigers in Southeast Asia. *Land* **9**, 415 (2020).

7. Ash, E., Cushman, S., Redford, T., Macdonald, D. & Kaszta, Ż. Tigers on the edge: mortality and landscape change dominate individual-based spatially-explicit simulations of a small tiger population. *Landsc. Ecol.* **37**, 3079–3102 (2022).

8. SERVIR-Mekong. *SERVIR–Mekong Regional Land Cover Monitoring System (RLCMS)*. https://rlcms-servir.adpc.net/en/landcover/ (2018).

9. OpenStreetMap. OpenStreetMap. www.openstreetmap.org (2019).

10. Kaszta, Ż. *et al.* Simulating the impact of Belt and Road initiative and other major developments in Myanmar on an ambassador felid, the clouded leopard, Neofelis nebulosa. *Landsc. Ecol.* **35**, 727–746 (2020).

11. Kaszta, Ż. *et al.* Integrating Sunda clouded leopard (Neofelis diardi) conservation into development and restoration planning in Sabah (Borneo). *Biol. Conserv.* **235**, 63–76 (2019).

12. Ash, E. *et al.* Estimating the density of a globally important tiger (Panthera tigris) population: Using simulations to evaluate survey design in Eastern Thailand. *Biol. Conserv.* **241**, 108349 (2020).

13. R Development Core Team. R: A language and environment for statistical computing. R Foundation for Statistical Computing. v.4.1.2. Vienna. (2021).

14. Ash, E. *et al.* Optimization of spatial scale, but not functional shape, affects the performance of habitat suitability models: A case study of tigers (Panthera tigris) in Thailand. *Landsc. Ecol.* **36**, 455–474 (2021).

15. Macdonald, D. W. *et al.* Multi-scale habitat selection modeling identifies threats and conservation opportunities for the Sunda clouded leopard (Neofelis diardi). *Biol. Conserv.* **227**, 92–103 (2018).

Supplementary Materials 2

1. Supplementary Methods

1.1 Resistance Surface

The process for developing the resistance surface was originally described by Ash et al. [1], which evaluated four approaches for predicting resistance: (1) A locally-developed, scale-optimized habitat suitability model from Ash et al. [2]; (2) A multivariate optimized resistance model developed by Reddy et al. [4], describing the relationship between tiger genetic distance and land cover variables in central India; (3) A resistance model for tigers in central India developed by Krishnamurthy et al. [5] using a scale-optimized path selection framework; and (4) An expert-derived resistance map based on reclassified 2018 SERVIR–Mekong Regional Land Cover Monitoring System (RLCMS [6]) data, incorporating major and minor roads [7]. Authors of the study determined that the expert map more broadly captured likely resistance to movement in the study landscape and utilized the expert-based resistance surface in their evaluation of landscape connectivity for tigers. This expert-based resistance layer was used in Ash et al. [8] to represent current estimates of landscape resistance to tiger movement and to act as a baseline for comparison of the effect of various landscape change scenarios.

The resistance surface used in this study defines resistance based on 2018 SERVIR–Mekong Regional Land Cover Monitoring System (RLCMS [6]) land cover data, classified into dense forest (resistance value of 1), scrub forest (20), agriculture/village matrix (50), reservoirs/surface water (80), and urban areas (100). Minor (30) and major roads (100 [7]) were also included (Table 1).

Table 1 Resistance values derived from reclassified land cover from 2018 SERVIR–Mekong Regional Land Cover Monitoring System(RLCMS [6]) and road network data [7], as described in Ash et al. [1].

Resistance Value (cost-distance unit / pixel)	Attributes / Land Cover Type
1	Dense Forest (*Evergreen Forest, Forest, Mixed Forest, Flooded Forest, Mangroves*)
20	Scrub Forest (*Shrubland, Grassland, Wetlands, Orchard/Plantation*)
30	Minor Roads
50	Agriculture-Village Matrix (*Rice, Cropland, Barren, Aquaculture*)
80	Reservoirs + Surface Water
100	Urban/Built-Up Areas, Major Roads

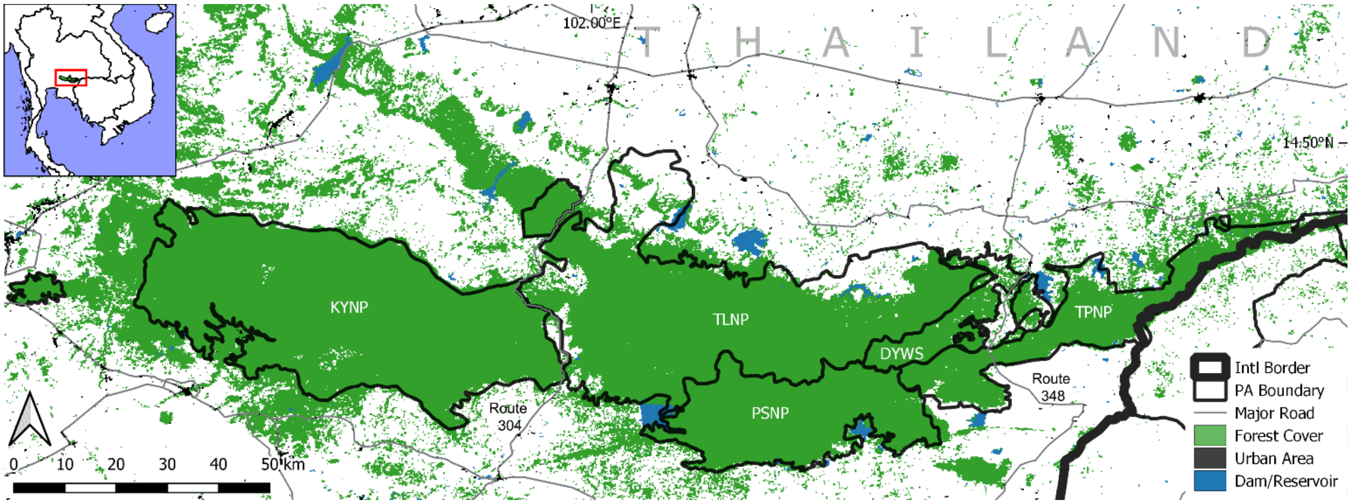

Fig. 1 Resistance surface of the study area representing current forest cover, reservoirs, urban areas, and major roads. Minor roads are not shown. From Ash et al. [8].

1.2. Mortality Function

In population connectivity studies, mortality may be implicitly incorporated through resistance surfaces [9], though the relationship between resistance surface and mortality may be unclear. The explicit incorporation of spatially-differential mortality risk in landscape-scale population modelling studies has been shown to dramatically affect conclusions pertaining to landscape connectivity and population persistence [1,8,10–13].

In order to account for heterogeneity in mortality risk across the study landscape, we incorporated a habitat-based mortality function (MH10) which emerged as the most plausible function among a series of functions assessed in Ash et al. [8]. This mortality function was developed as a product of predicted tiger habitat suitability, landscape resistance, and protected area status with values representing the probability of a tiger that has dispersed to a given pixel dying before reproducing. Forested areas of low resistance (1-20) were adjusted by a habitat suitability prediction surface from layer from Ash et al. [2], inverted and rescaled between 0-20 percent mortality probability. In effect, the highest quality habitat would have a low probability of mortality (~2%-8%) with a max mortality probability of 20% (corresponding with a mean ~80% annual survival documented in well-protected forest in Western Thailand; [14]). Outside PAs, the mortality risk in forest areas was doubled, with most unprotected forest corresponding to roughly 40% mortality probability before further adjustments. Similar to Kaszta et al. [13], long-distance effects of anthropogenic structures, such as roads, dams/reservoirs, and urban areas were included as buffers, with mortality probability declining logarithmically up to the distance of 17,400m for roads and dams, and over 5,000m for urban areas.

2. Supplementary Results

Here, we discuss the relative effect of translocations in terms of the number of simulations in which population, allelic richness, and heterozygosity values were (1) stable/increased, (2) declined, and (3) zero (extinction) across various scenarios.

2.1 Population

Simulations in which no translocations occurred (base scenario) produced stable or increasing populations in 50.3% of simulations, declining populations in 26.7% of simulations, and 23% resulted in extinction (Table 2).

Comparing the translocation of new individuals into the population (TSEX), simulations produced populations that were stable or increasing in 50.0% (-0.3%; TF0M2) to 50.7% (+0.4%; TF0M1) of simulations, with extinction rates of 22.1% (-0.9%; TF0M1) to 22.3% (-0.7%; TF0M4; Table 2). In contrast, in simulations translocations of females, populations were stable or increased in 58% (+7.7%; TF1M0) to 70.5% (+20.2%; TF4M0) of simulations and had extinction rates ranging from 10.5% (-12.5%; TF4M0) to 17.4% (-5.6%; TF1M0). Two scenarios included equal numbers of males and females translocated into the population (TF1M1 and TF2M2). These simulations produced stable or increasing populations in 57.1% (+6.8%; TF1M1) and 63.6% (+13.3%; TF2M2) of simulations and extinction rates of 13.5% (-9.5%; TF2M2) to 17% (-6.0%; TF1M1).

When comparing differences in the number of translocations (TFRQ), simulations resulted in stable or increasing *N* from 52.2% (+1.9%; TFRQ1) to 69.5% (+19.2%; TFRQ20) of simulations, with extinction rates ranging from 10.5% (-12.5%; TFRQ20) to 21.5% (-1.5%; TFRQ1).

Comparing differences in mean *N* at generation 20 between different translocation mortality thresholds (TMOR), the rate of stable or increasing populations in these scenarios ranged from 54.6% (+4.3%; TMOR75) to 60.6% (+10.3%; TMOR25) with extinction rates from 15.8% (-7.2%; TMOR25) to 19.4% (-3.6%; TMOR75).

Differences in the effect of sex and number of individuals translocated into the population (TSEX) are also evident when evaluating mean *N* in conjunction with the frequency of translocations (TFRQ; Table 3). In male-only translocation scenarios, simulations in which populations were stable or increasing occurred in 48.3% (-2%; TF0M2-TFRQ3) to 50.9% (+0.4%; TF0M1-TFRQ20) of simulations, with extinction rates ranging from 19.1% (TF0M4-TFRQ20) to 24.3% (TF0M2-TFRQ3). Comparing number of individuals and frequency of translocations of females, simulations resulted in stable or increasing populations in 53.2% (+2.9%; TF1M0-TFRQ1) to 94.7% (+44.4%; TF4M0-TFRQ20) of simulations with extinction rates ranging from 0.5% (-22.5%; TF4M0-TFRQ20) to 21.1% (-1.9%; TF1M0-TFRQ3).

2.2 Effect of translocations on genetic trajectory

We evaluate the simulated genetic trajectory of the DPKY population in two ways, based on allelic richness (*AR*) and observed heterozygosity (*Ho*). In simulations in which no translocations occurred *AR* and *Ho* values declined or were zero due to extinction in 99.1% and 99.7% of simulations, respectively (Table 2).

Comparing adjustment of starting mean alleles per locus for DPKY individuals (APL), declines in *AR* and *Ho* were more frequent in simulations where DPKY individuals had a higher initial average number of alleles per locus. *AR* was lower in 51.9% of simulations in APL2 compared to 82.1% in APL4 and *Ho* was lower in 75.8% of APL2 simulations compared to 93.2% in APL4 (Table 2).

Among scenarios in which the percentage of DPKY alleles shared with WEFCOM individuals were varied (SA%). Approximately, 57.4% (-41.7%; SA25) to 63.2% (-35.9%; SA75) of simulations resulted in *AR* declines while 79.1% (-20.6%; SA25) to 84.8% (-14.9%; SA75) resulted in declines of *Ho* (Table 2).

When evaluating differences in genetic trajectories based on sex and number of translocations (TSEX), among male-only translocation scenarios, declines in *AR* were observed in 59.1% (-40% from base scenario; TF0M4) to 79.7% (-19.4%; TF0M1) of simulations while declines in *Ho* were observed 77.9% (-21.8%; TF0M4) and 93.9% (-5.9%; TF0M1) of simulations (Table 2). Across scenarios involving female-only translocations, declines in *AR* were observed in 40.4% (-58.7%; TF4M0) to 70.2% (-28.9%; TF1M0) of simulations while declines in *Ho* were observed 69% (-30.7%; TF4M0) to 92.0% (-7.7%; TF1M0) of simulations. In mixed-sex translocation scenarios, *AR* declines were observed in 61.4% (-37.7%) of TF1M1 simulations and 45.7% (-53.4%) of TF2M2 while declines in *Ho* were observed in 83.7% (-16%) and 70.6% (-29.1%) of simulations in these respective scenarios.

When comparing the frequency in which individuals were translocated (TFRQ), declines in *AR* were observed in 24% (-75.1%; TFRQ20) to 88% (-11.1%; TFRQ1) of simulations and declines in *Ho* were observed in 47.7% (-52%; TFRQ20) to 98.4% (-1.3%; TFRQ1) of simulations (Table 2).

Differences in genetic trajectories with adjustment of mortality probability of translocated individuals (TMOR) were moderate. Declines in *AR* were observed in 50.0% (-49.1%; TMOR25) to 72% (-29.1%; TMOR75) of simulations while declines in *Ho* were observed in 74.1% (-25.6%; TMOR25) to 90.4% (-9.3%; TMOR75) of simulations (Table 2).

Evaluating the effect of sex and number of individuals translocated into the population (TSEX), in conjunction with the frequency of translocations (TFRQ; Table 3), in male-only translocation scenarios, declines in *AR* were observed in 29.0% (-70.1%; TF0M4-TFRQ20) to 94.0% (-5.1%; TF0M1-TFRQ1) of simulations while declines in *Ho* occurred in 41.3% (-58.4%; TF0M4-TFRQ20) to 99.2% (-0.5%; TF0M1-TFRQ1) of simulations. In female-only scenarios, the percentage of simulations resulting in lower *AR* and *Ho* values ranged from 1.7% (-97.4%; TF4M0-TFRQ20) to 92.4% (-6.7%; TF1M0-TFRQ1) and 19.2% (-80.5%; TF4M0-TFRQ20) to 99.6% (-0.1%; TF1M0-TFRQ1), respectfully. Lastly, comparing scenarios in which equal numbers of males and females were translocated, declines in *AR* were observed in 6.2% (-92.9%; TF2M2-TFRQ20) to 90.0% (-9.1%; TF1M1-TFRQ1) of simulations while declines in *Ho* occurred in 21.4% (-78.2%; TF2M2-TFRQ20) to 99.1% (-0.6%; TF1M1-TFRQ1) of simulations.

2.3 Variance of Population and Genetic Trajectories

Overall, population trajectories even in simulations using the same parameters, were highly variable, producing both extinctions and relative increases by the final timestep. The coefficient of variation (CV) for *N* ranged from 14.67 (TF4M0-APL3-SA25-TFRQ20-TMOR25) to 112.87 (TF0M2-APL4-SA50-TFRQ3-TMOR75). For *AR*, CV ranged from 5.58 (TF4M0-APL3-SA75-TFRQ20-TMOR25) to 78.74 (TF0M4-APL4-SA25-TFRQ2-TMOR50) while *Ho* CV ranged from 4.20 (TF4M0-APL4-SA25-TFRQ20-TMOR25) to 80.48 (TF0M1-APL2-SA75-TFRQ10-TMOR75).

Supplementary Results Tables

Table 2. Comparison of percentage of simulations resulting in stable/increasing values, declining values (>0), and extinctions across population (*N*), allele richness (*AR*), and heterozygosity (*Ho*).

		N			*AR*			*Ho*		
		Stable/ Increasing	Declines	Extinctions	Stable/ Increasing	Declines	Extinctions	Stable/ Increasing	Declines	Extinctions
APL										
	APL2	56.5	25.1	18.4	48.0	33.5	18.4	24.1	57.4	18.4
	APL3	56.7	25.0	18.3	32.3	49.4	18.3	13.5	68.2	18.3
	APL4	56.4	25.1	18.6	17.9	63.5	18.6	6.8	74.6	18.6
SA%										
	SA0	50.3	26.7	23.0	0.9	76.1	23.0	0.3	76.7	23.0
	SA25	58.1	24.6	17.3	42.6	40.1	17.3	20.9	61.8	17.3
	SA50	57.7	24.8	17.5	40.1	42.4	17.5	18.1	64.4	17.5
	SA75	57.9	24.6	17.5	36.8	45.7	17.5	15.2	67.3	17.5
TSEX										
	TF0M0	50.3	26.7	23.0	0.9	76.1	23.0	0.3	76.7	23.0
	TF0M1	50.7	27.2	22.1	20.3	57.6	22.1	6.1	71.7	22.1
	TF0M2	50.0	27.8	22.2	30.4	47.4	22.2	13.1	64.6	22.2
	TF0M4	50.3	27.4	22.3	40.9	36.8	22.3	22.1	55.6	22.3
	TF1M0	58.0	24.6	17.4	29.8	52.8	17.4	8.0	74.6	17.4
	TF2M0	63.0	22.8	14.2	45.0	40.8	14.2	18.4	67.3	14.2
	TF4M0	70.5	19.0	10.5	59.5	29.9	10.5	30.9	58.5	10.5
	TF1M1	57.1	25.9	17.0	38.5	44.4	17.0	16.3	66.7	17.0
	TF2M2	63.6	22.9	13.5	54.2	32.2	13.5	29.4	57.1	13.5
TFRQ										
	TFRQ0	50.3	26.7	23.0	0.9	76.1	23.0	0.3	76.7	23.0
	TFRQ1	52.2	26.3	21.5	11.9	66.5	21.5	1.5	76.9	21.5
	TFRQ2	53.1	26.5	20.5	23.9	55.6	20.5	4.4	75.1	20.5
	TFRQ3	54.2	26.3	19.5	30.8	49.7	19.5	8.2	72.3	19.5
	TFRQ4	55.8	26.1	18.1	38.2	43.7	18.1	12.4	69.5	18.1
	TFRQ10	62.6	22.9	14.5	58.1	27.4	14.5	29.5	56.0	14.5
	TFRQ20	69.5	20.1	10.5	76.0	13.5	10.5	52.4	37.2	10.5
TMOR										
	TMOR0	50.3	26.7	23.0	0.9	76.1	23.0	0.3	76.7	23.0
	TMOR25	60.6	23.6	15.8	50.0	34.2	15.8	26.0	58.3	15.8
	TMOR50	58.5	24.4	17.1	41.5	41.4	17.1	18.6	64.3	17.1
	TMOR75	54.6	26.0	19.4	27.9	52.6	19.4	9.6	71.0	19.4

Table 3. Comparison of percentage of simulations resulting in stable/increasing values, declining values (>0), and extinctions across population (*N*), allele richness (*AR*), and heterozygosity (*Ho*), distinguished by number/sex of translocated individuals (TSEX) and frequency of translocations (TFRQ).

			N			*AR*			*Ho*	
TSEX	TFRQ	Stable/ Increasing	Declines	Extinctions	Stable/ Increasing	Declines	Extinctions	Stable/ Increasing	Declines	Extinctions
TF0M0	TFRQ0	50.3%	26.7%	23.0%	0.9%	76.1%	23.0%	0.3%	76.7%	23.0%
Male Translocations										
TF0M1	TFRQ1	50.5%	26.8%	22.7%	6.0%	71.3%	22.7%	0.9%	76.5%	22.7%
TF0M1	TFRQ2	50.6%	27.5%	21.9%	9.8%	68.3%	21.9%	1.4%	76.7%	21.9%
TF0M1	TFRQ3	50.6%	27.3%	22.0%	14.5%	63.5%	22.0%	2.3%	75.6%	22.0%
TF0M1	TFRQ4	50.8%	27.2%	22.0%	17.4%	60.6%	22.0%	3.6%	74.4%	22.0%
TF0M1	TFRQ10	50.6%	26.2%	23.2%	28.3%	48.5%	23.2%	8.1%	68.7%	23.2%
TF0M1	TFRQ20	50.9%	28.1%	21.0%	45.8%	33.2%	21.0%	20.6%	58.4%	21.0%
TF0M2	TFRQ1	50.9%	27.0%	22.1%	10.3%	67.6%	22.1%	1.1%	76.8%	22.1%
TF0M2	TFRQ2	48.7%	27.3%	24.0%	17.0%	59.0%	24.0%	2.9%	73.1%	24.0%
TF0M2	TFRQ3	48.3%	27.4%	24.3%	22.8%	52.9%	24.3%	5.7%	70.0%	24.3%
TF0M2	TFRQ4	50.2%	27.9%	21.9%	26.9%	51.2%	21.9%	7.7%	70.4%	21.9%
TF0M2	TFRQ10	51.6%	27.2%	21.1%	43.7%	35.1%	21.1%	20.1%	58.7%	21.1%
TF0M2	TFRQ20	50.3%	29.9%	19.9%	61.6%	18.5%	19.9%	41.3%	38.8%	19.9%
TF0M4	TFRQ1	52.2%	25.7%	22.1%	13.4%	64.5%	22.1%	2.1%	75.8%	22.1%
TF0M4	TFRQ2	49.0%	26.9%	24.2%	26.6%	49.2%	24.2%	6.6%	69.2%	24.2%
TF0M4	TFRQ3	49.2%	27.6%	23.2%	33.8%	43.0%	23.2%	11.2%	65.6%	23.2%
TF0M4	TFRQ4	50.3%	26.8%	22.9%	42.4%	34.7%	22.9%	17.2%	59.9%	22.9%
TF0M4	TFRQ10	50.4%	27.2%	22.3%	58.3%	19.4%	22.3%	36.9%	40.7%	22.3%
TF0M4	TFRQ20	50.7%	30.1%	19.1%	71.0%	9.9%	19.1%	58.7%	22.2%	19.1%
Female Translocations										
TF1M0	TFRQ1	54.2%	25.1%	20.7%	7.6%	71.7%	20.7%	0.4%	78.9%	20.7%
TF1M0	TFRQ2	53.4%	26.3%	20.3%	14.5%	65.2%	20.3%	1.3%	78.4%	20.3%
TF1M0	TFRQ3	53.2%	25.7%	21.1%	17.7%	61.2%	21.1%	1.8%	77.1%	21.1%
TF1M0	TFRQ4	54.9%	26.7%	18.4%	25.1%	56.5%	18.4%	3.0%	78.6%	18.4%
TF1M0	TFRQ10	61.5%	23.2%	15.3%	44.0%	40.7%	15.3%	12.7%	72.0%	15.3%
TF1M0	TFRQ20	70.7%	20.6%	8.6%	69.6%	21.8%	8.6%	29.0%	62.4%	8.6%
TF2M0	TFRQ1	53.4%	26.9%	19.7%	12.8%	67.5%	19.7%	1.5%	78.8%	19.7%
TF2M0	TFRQ2	55.1%	26.3%	18.6%	25.3%	56.1%	18.6%	3.8%	77.6%	18.6%
TF2M0	TFRQ3	57.3%	25.5%	17.2%	33.4%	49.4%	17.2%	6.0%	76.9%	17.2%
TF2M0	TFRQ4	57.9%	25.9%	16.1%	42.0%	41.9%	16.1%	9.6%	74.2%	16.1%
TF2M0	TFRQ10	70.9%	20.0%	9.1%	68.6%	22.3%	9.1%	30.8%	60.1%	9.1%
TF2M0	TFRQ20	83.1%	12.4%	4.6%	88.0%	7.4%	4.6%	59.0%	36.4%	4.6%
TF4M0	TFRQ1	54.6%	25.1%	20.3%	18.6%	61.1%	20.3%	1.9%	77.8%	20.3%
TF4M0	TFRQ2	59.1%	24.9%	16.0%	41.2%	42.7%	16.0%	7.8%	76.2%	16.0%
TF4M0	TFRQ3	63.8%	24.3%	11.9%	51.2%	36.9%	11.9%	15.9%	72.1%	11.9%
TF4M0	TFRQ4	67.9%	21.9%	10.3%	62.5%	27.2%	10.3%	26.1%	63.6%	10.3%
TF4M0	TFRQ10	82.9%	13.0%	4.2%	85.5%	10.3%	4.2%	52.9%	43.0%	4.2%
TF4M0	TFRQ20	94.7%	4.7%	0.5%	98.3%	1.2%	0.5%	80.8%	18.7%	0.5%
Mixed Translocations										
TF1M1	TFRQ1	50.3%	26.4%	23.3%	10.0%	66.7%	23.3%	0.9%	75.8%	23.3%
TF1M1	TFRQ2	52.3%	27.4%	20.3%	19.3%	60.4%	20.3%	3.4%	76.3%	20.3%
TF1M1	TFRQ3	52.6%	27.1%	20.4%	28.1%	51.5%	20.4%	7.6%	72.0%	20.4%
TF1M1	TFRQ4	55.4%	26.0%	18.6%	35.4%	46.0%	18.6%	9.9%	71.5%	18.6%
TF1M1	TFRQ10	61.6%	26.0%	12.4%	58.5%	29.1%	12.4%	24.9%	62.7%	12.4%
TF1M1	TFRQ20	70.3%	22.5%	7.2%	79.9%	12.9%	7.2%	51.0%	41.8%	7.2%
TF2M2	TFRQ1	51.3%	27.4%	21.3%	16.8%	61.9%	21.3%	3.5%	75.2%	21.3%
TF2M2	TFRQ2	56.3%	25.3%	18.4%	37.5%	44.1%	18.4%	8.1%	73.6%	18.4%
TF2M2	TFRQ3	58.2%	25.9%	15.9%	45.3%	38.8%	15.9%	15.0%	69.1%	15.9%
TF2M2	TFRQ4	59.2%	26.1%	14.7%	53.9%	31.4%	14.7%	21.6%	63.7%	14.7%
TF2M2	TFRQ10	71.1%	20.6%	8.3%	78.0%	13.8%	8.3%	49.5%	42.3%	8.3%
TF2M2	TFRQ20	85.1%	12.1%	2.8%	93.8%	3.4%	2.8%	78.6%	18.7%	2.8%

Supplementary Results Figures

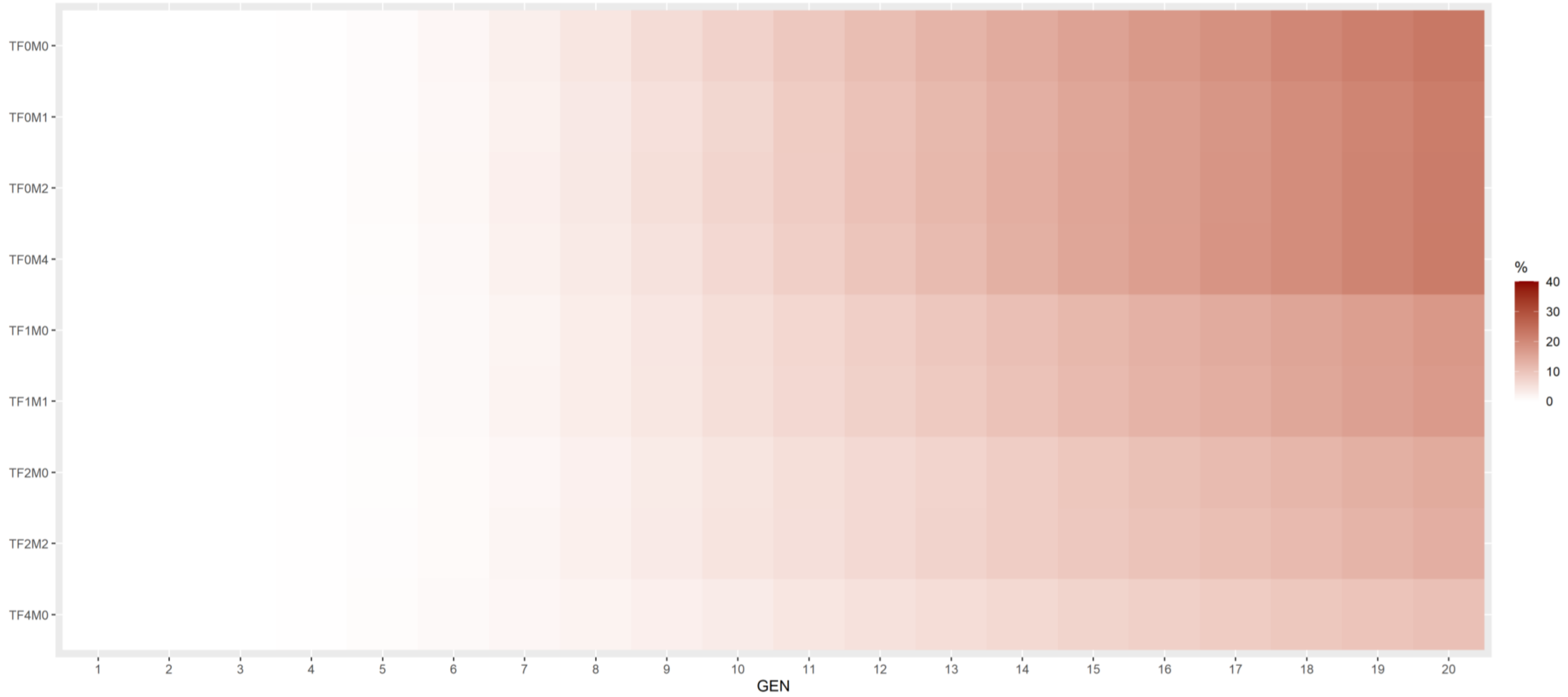

Fig. 2 Comparison of probability of extinction (percentage of simulations in which *N*=0) from generation 1 to 20 across number/sex of translocated individuals (TSEX).

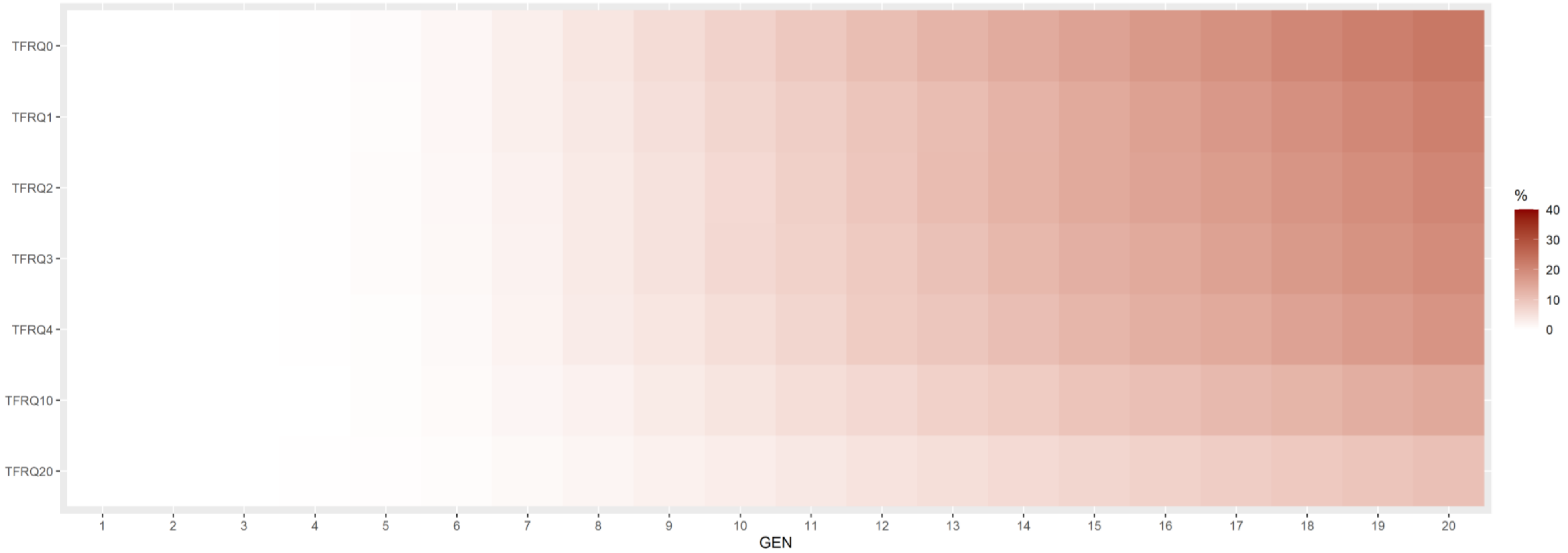

Fig. 3 Comparison of probability of extinction (percentage of simulations in which *N*=0) from generation 1 to 20 across frequency of translocations (TFRQ).

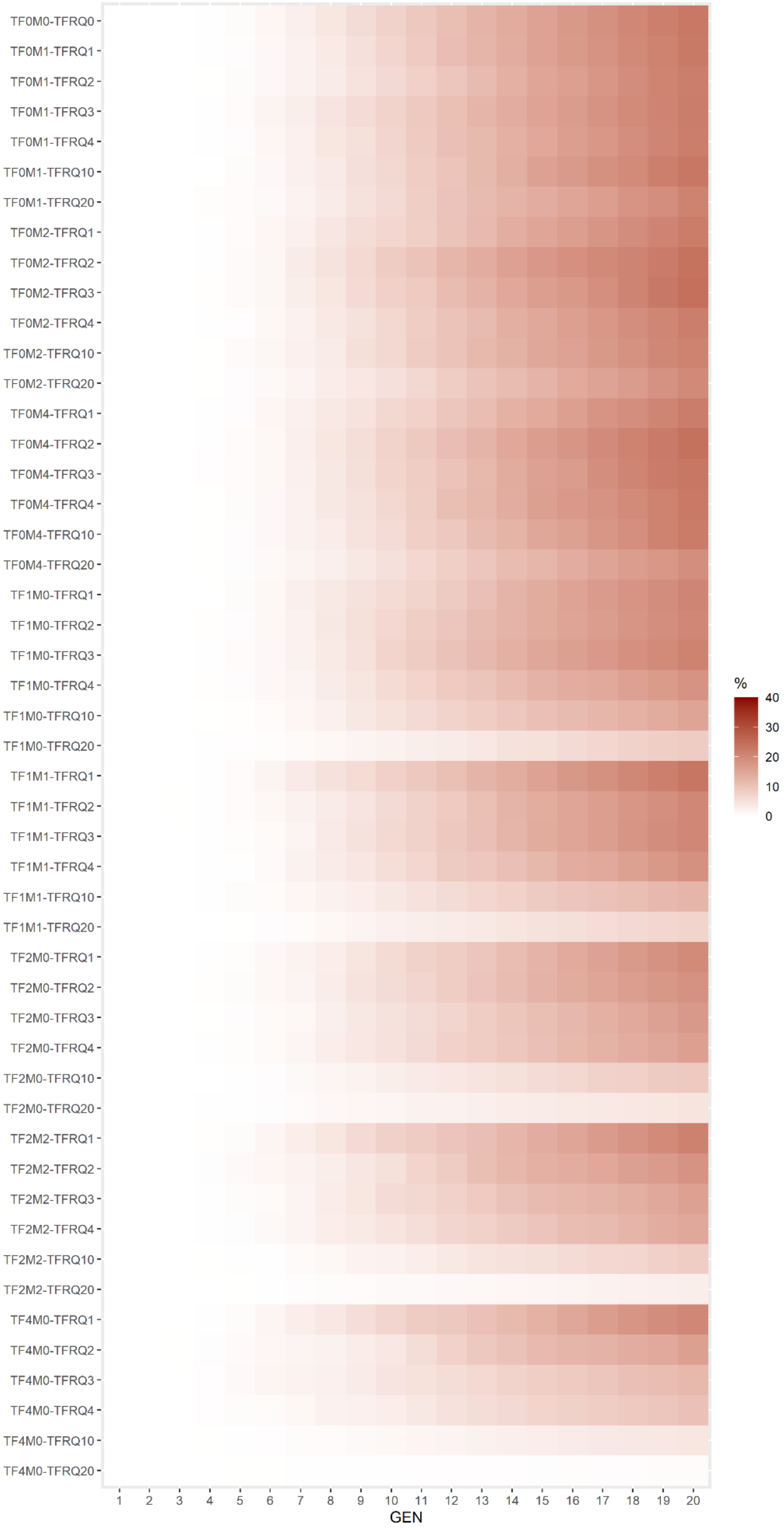

Fig. 4 Comparison of probability of extinction (percentage of simulations in which *N*=0) from generation 1 to 20 across number/sex of translocated individuals (TSEX) and frequency of translocations (TFRQ).

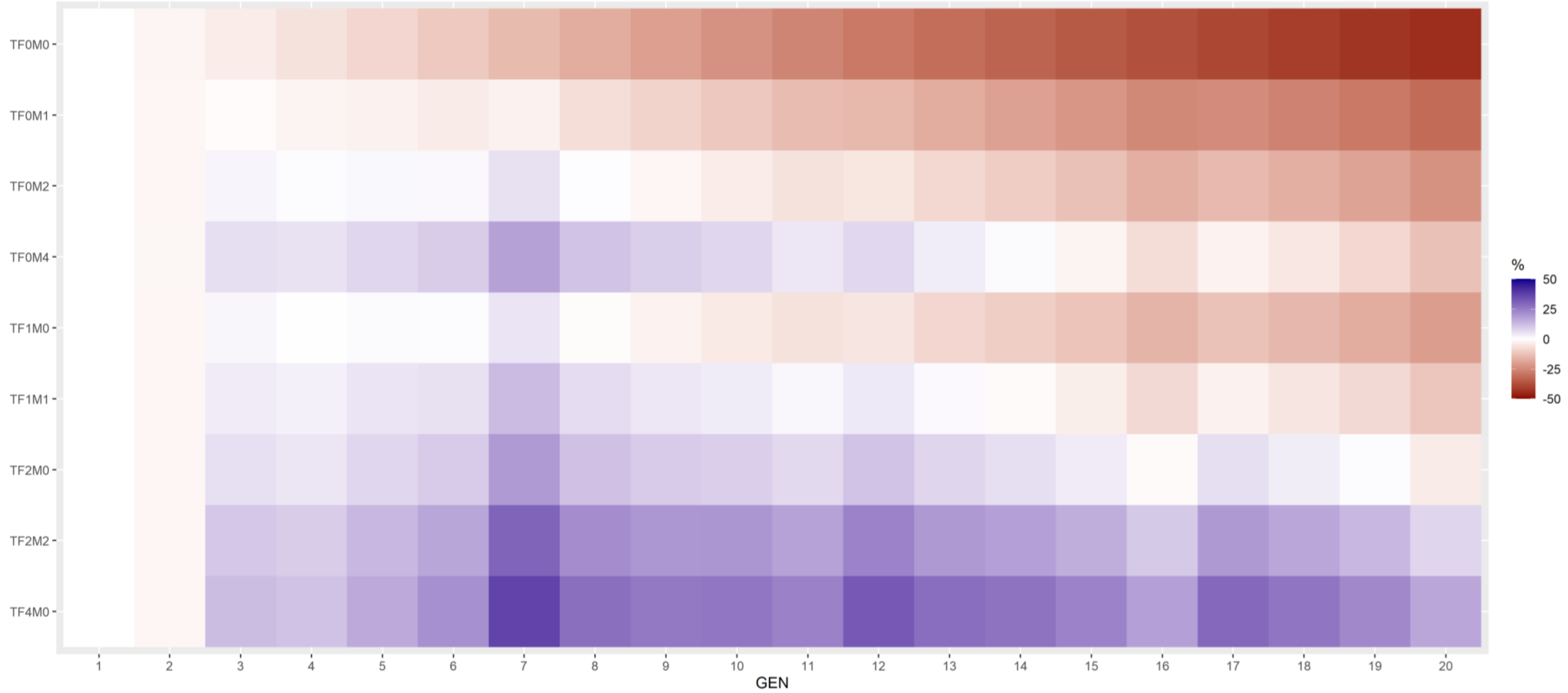

Fig. 5 Percentage change in allelic richness (*AR*) relative to generation 1 across number/sex of translocated individuals (TSEX).

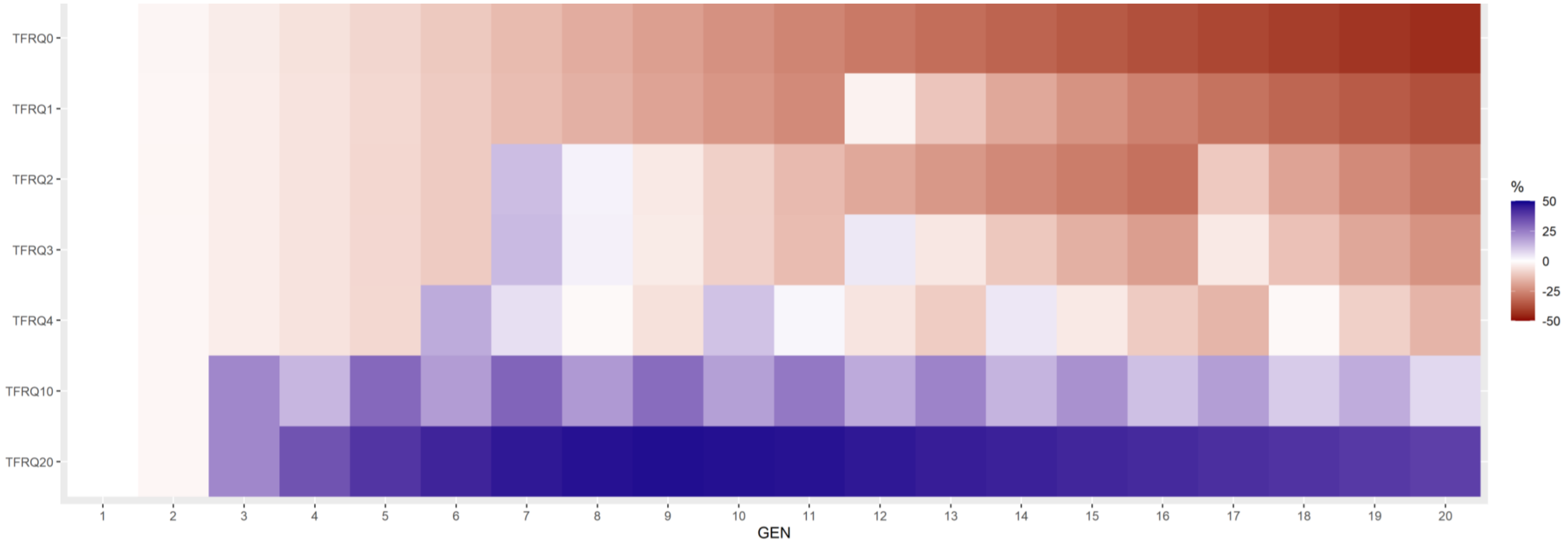

Fig. 6 Percentage change in allelic richness (*AR*) relative to generation 1 across frequency of translocations (TFRQ).

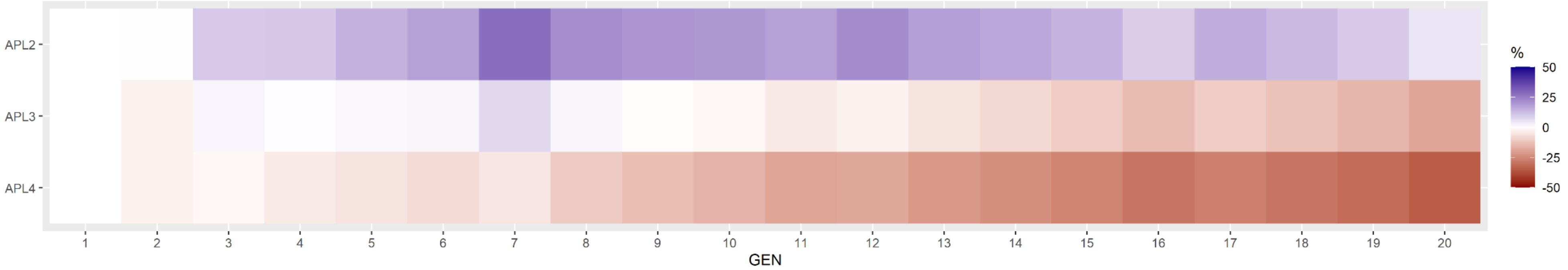

Fig. 7. Percentage change in allelic richness (*AR*) relative to generation 1 across different starting average alleles per locus for individuals in DPKY (APL).

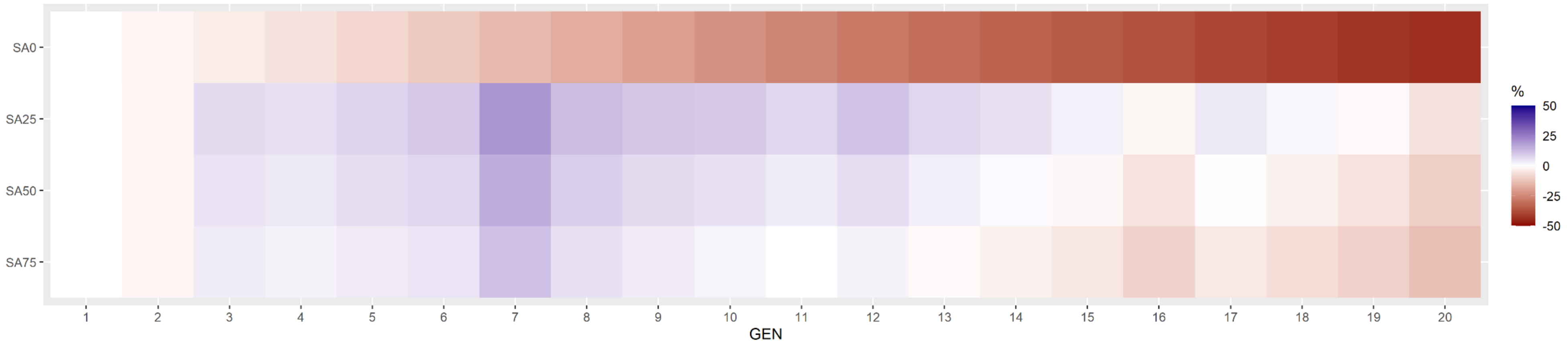

Fig. 8 Percentage change in allelic richness (*AR*) relative to generation 1 across scenarios in which the percentage of DPKY alleles shared with WEFCOM individuals were varied (SA%).

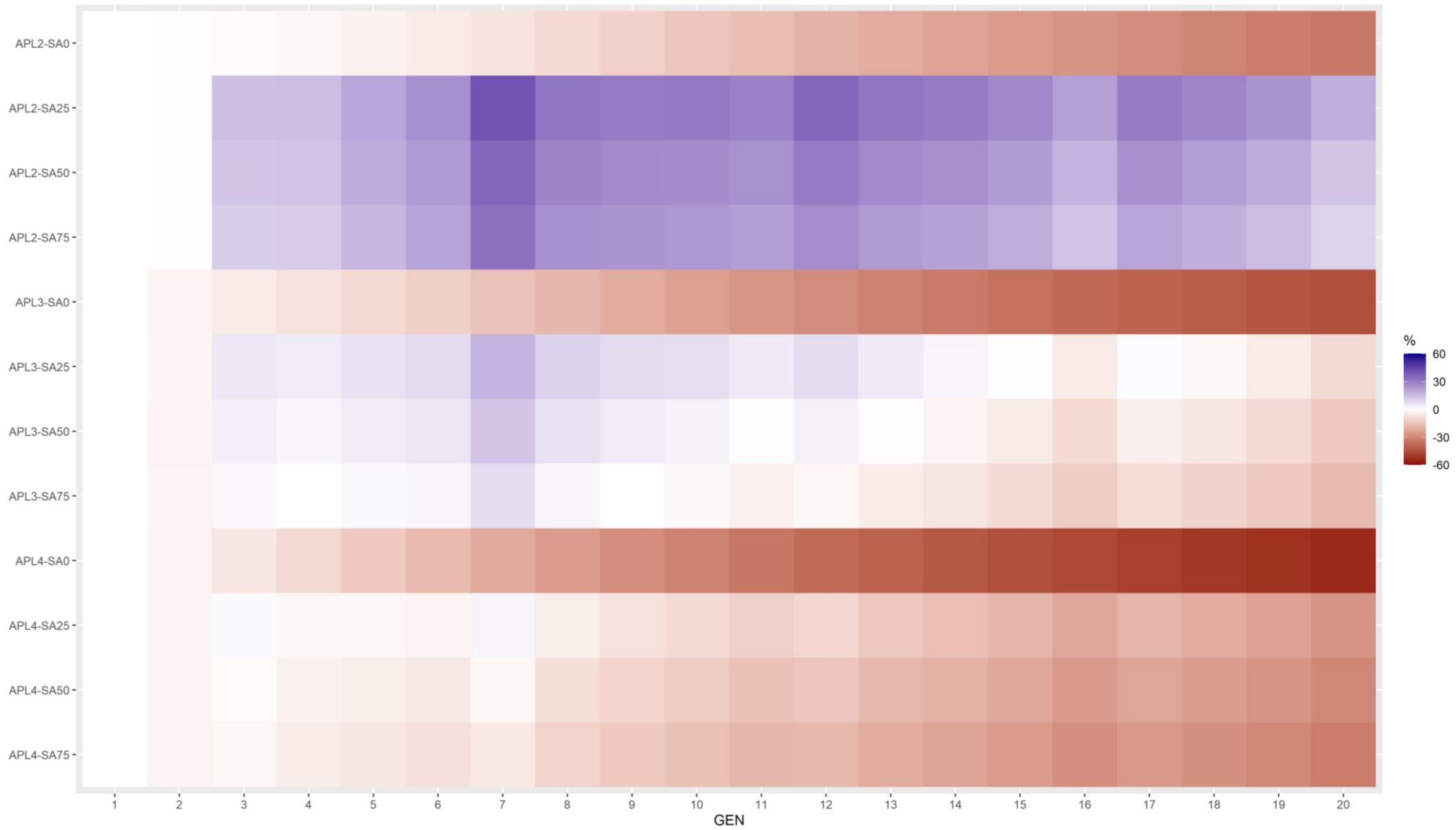

Fig. 9 Percentage change in allelic richness (*AR*) relative to generation 1 across different starting average alleles per locus for individuals in DPKY (APL) and scenarios in which the percentage of DPKY alleles shared with WEFCOM individuals were varied (SA%).

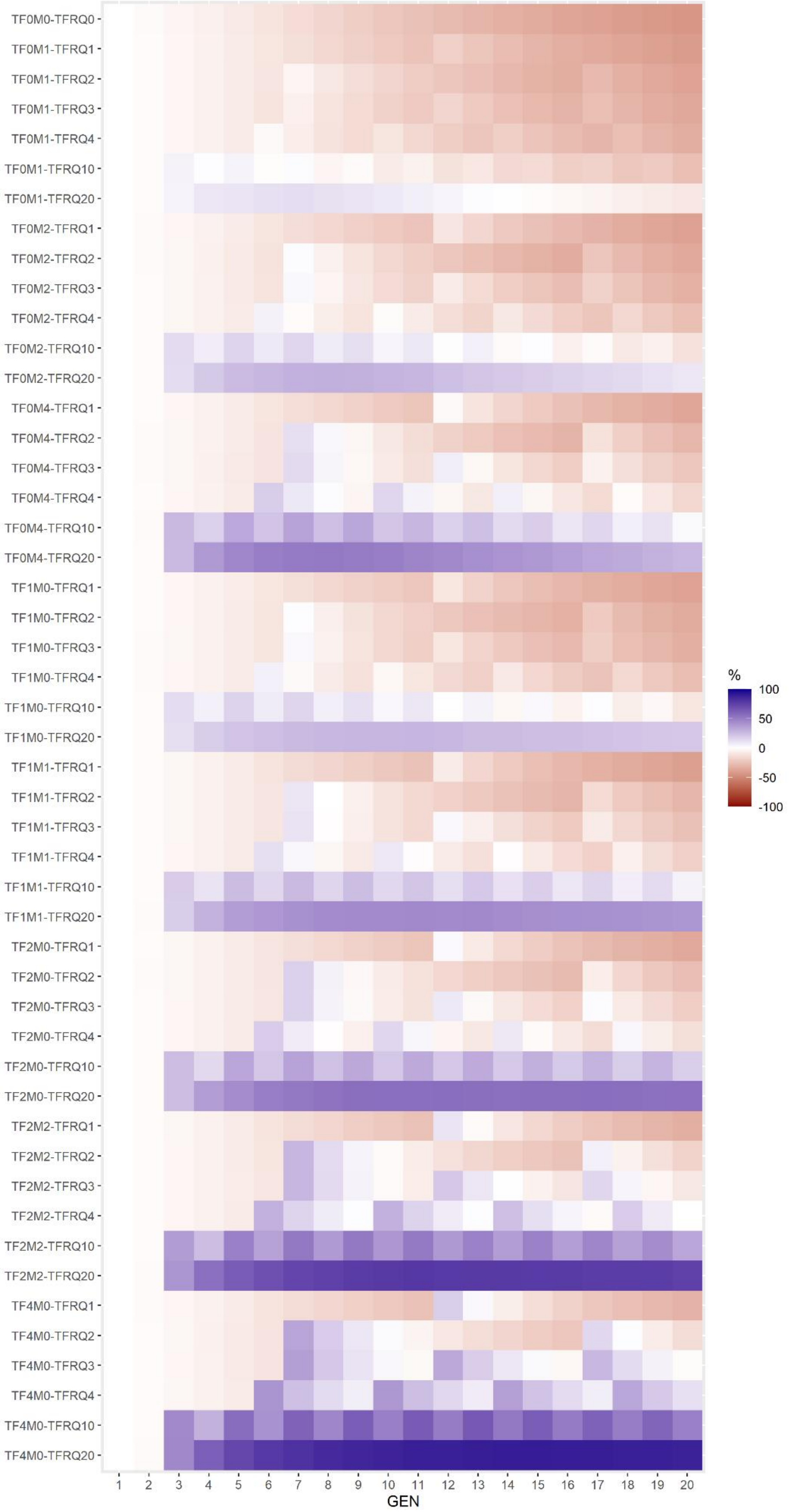

Fig. 10 Percentage change in allelic richness (*AR*) relative to generation 1 across number/sex of translocated individuals (TSEX) and frequency of translocations (TFRQ).

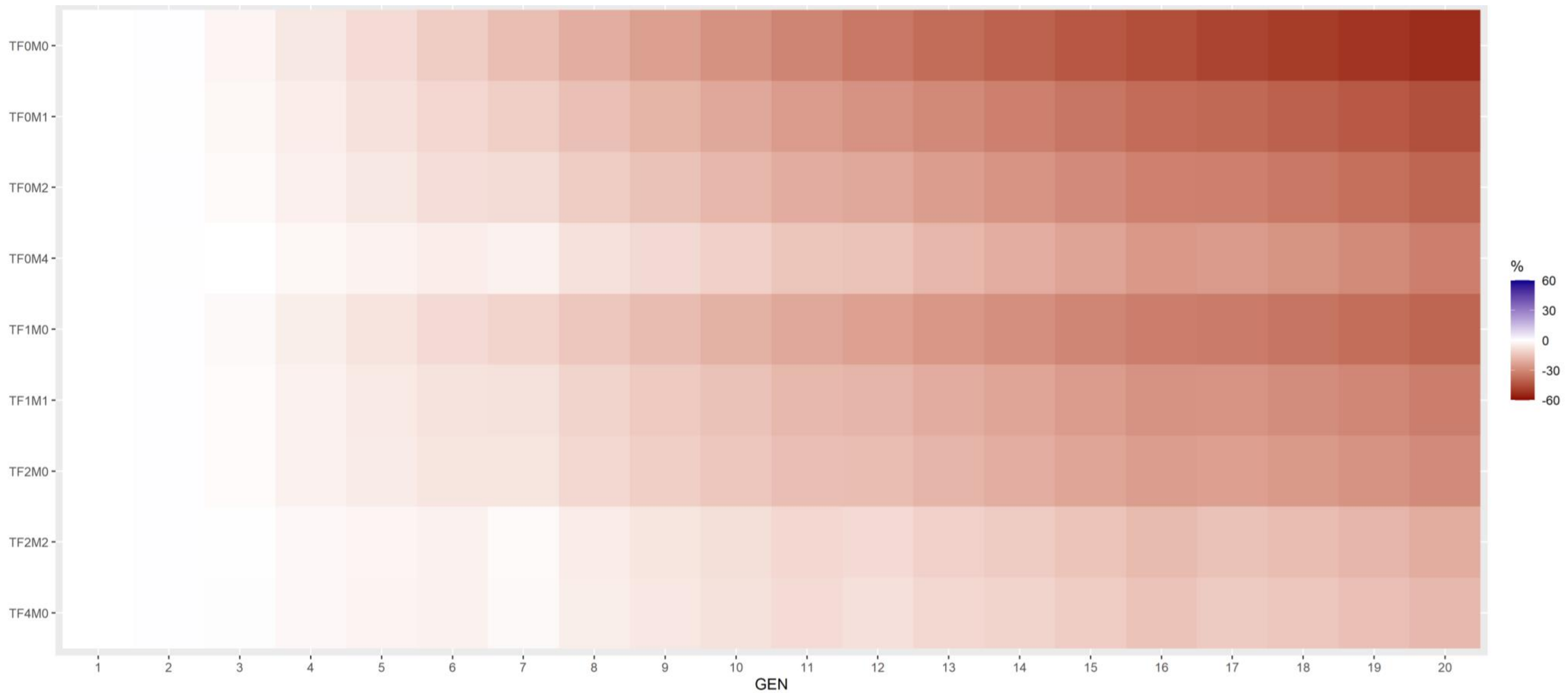

Fig. 11 Percentage change in heterozygosity (*Ho*) relative to generation 1 across number/sex of translocated individuals (TSEX).

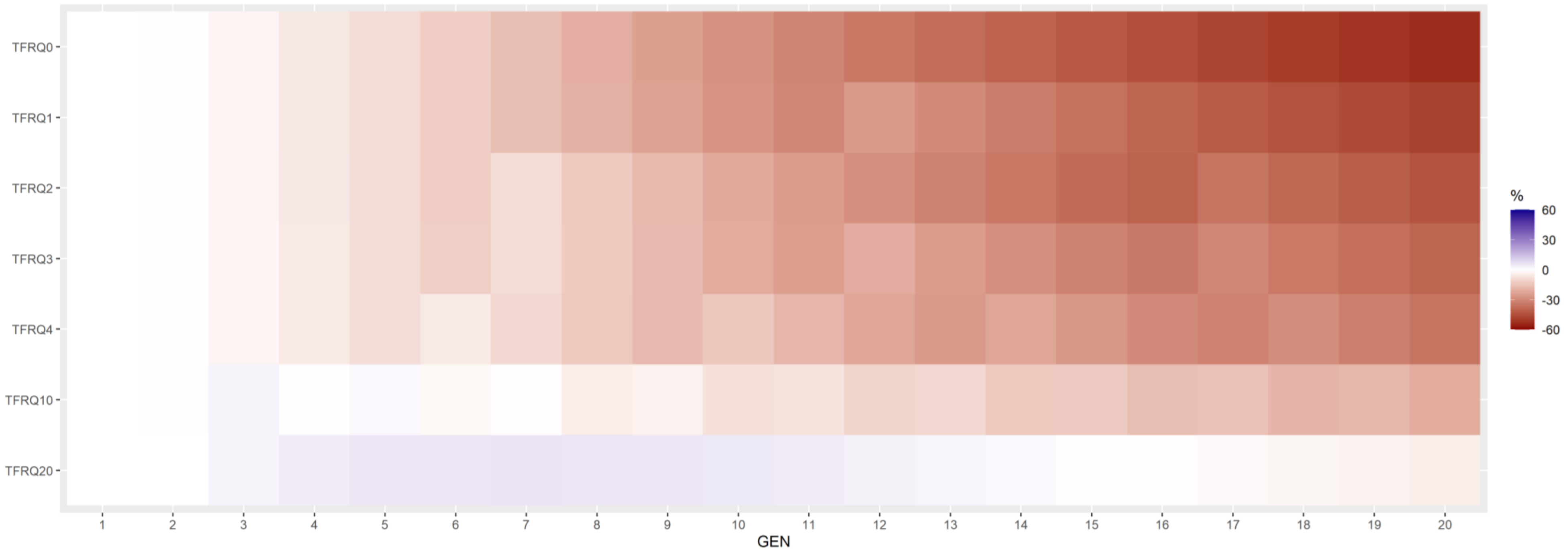

Fig. 12 Percentage change in heterozygosity (*Ho*) relative to generation 1 across frequency of translocations (TFRQ).

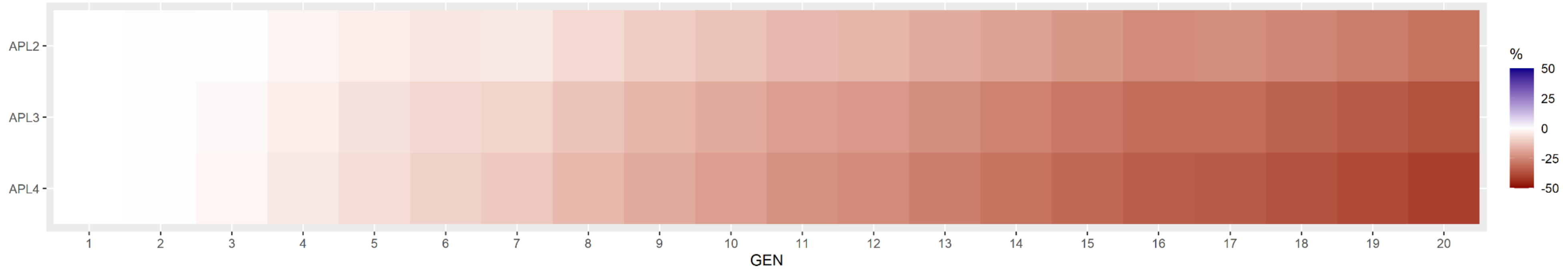

Fig. 13 Percentage change in heterozygosity (*Ho*) relative to generation 1 across different starting average alleles per locus for individuals in DPKY (APL).

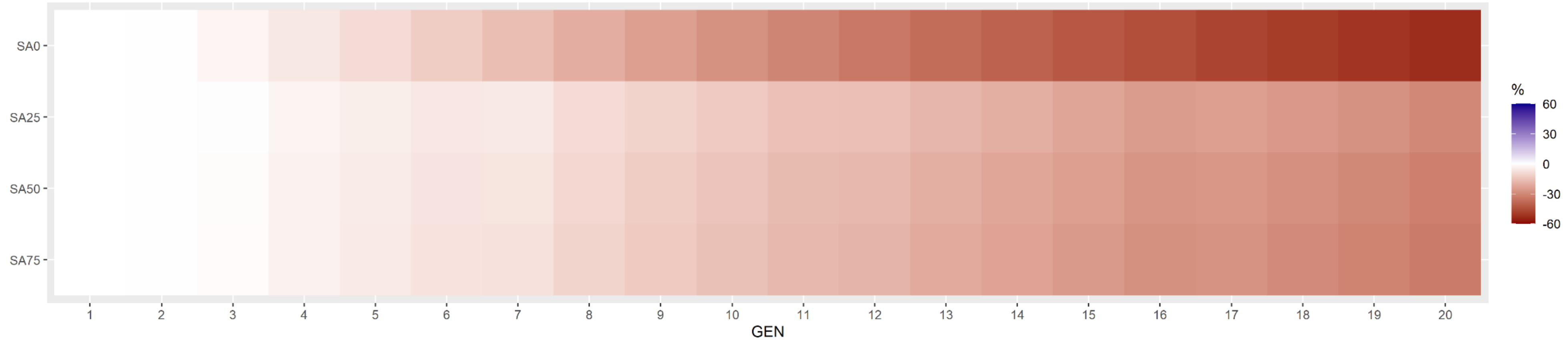

Fig. 14 Percentage change in heterozygosity (*Ho*) relative to generation 1 across scenarios in which the percentage of DPKY alleles shared with WEFCOM individuals were varied (SA%).

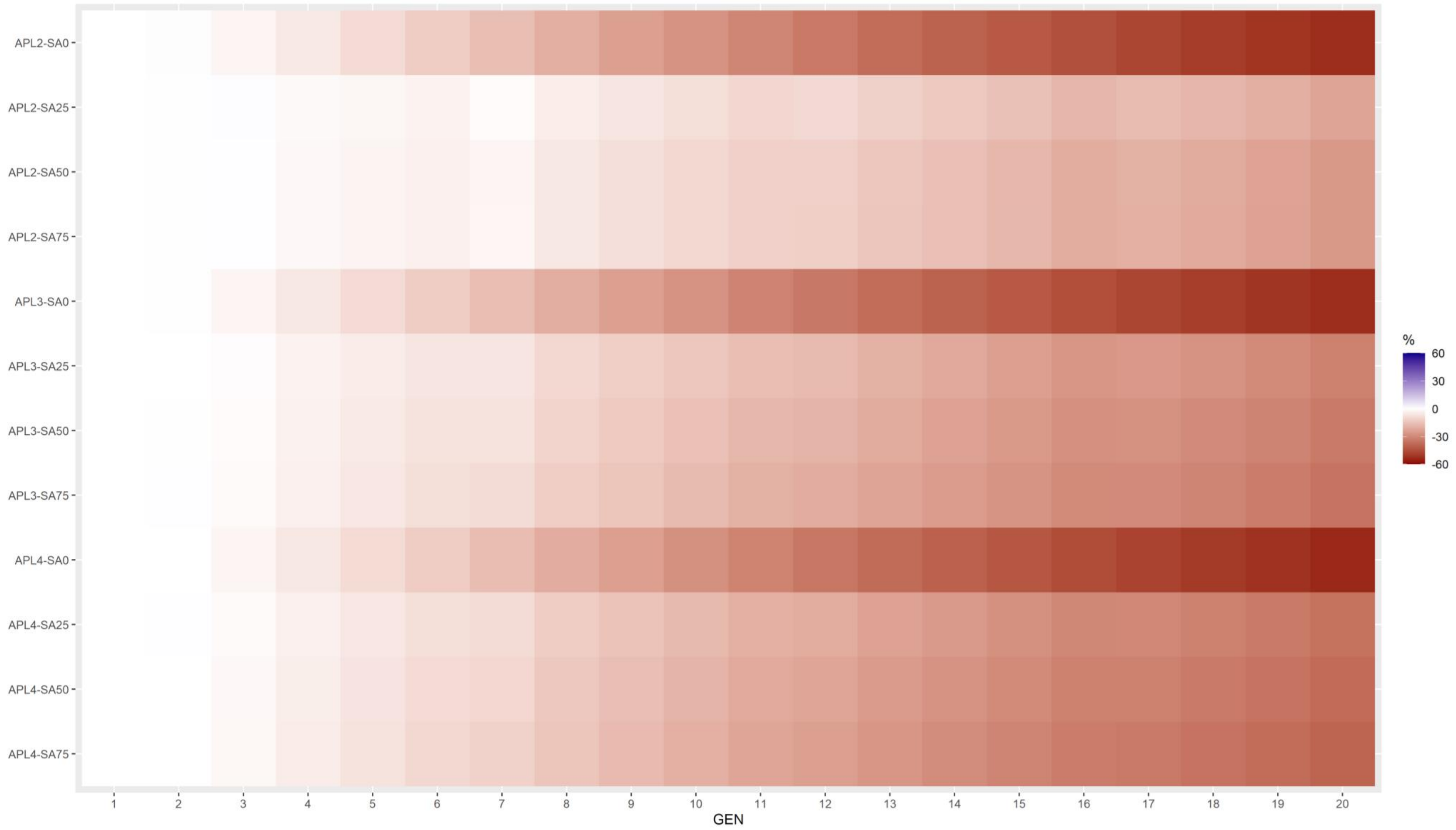

Fig. 15 Percentage change in heterozygosity (*Ho*) relative to generation 1 across different starting average alleles per locus for individuals in DPKY (APL) and scenarios in which the percentage of DPKY alleles shared with WEFCOM individuals were varied (SA%).

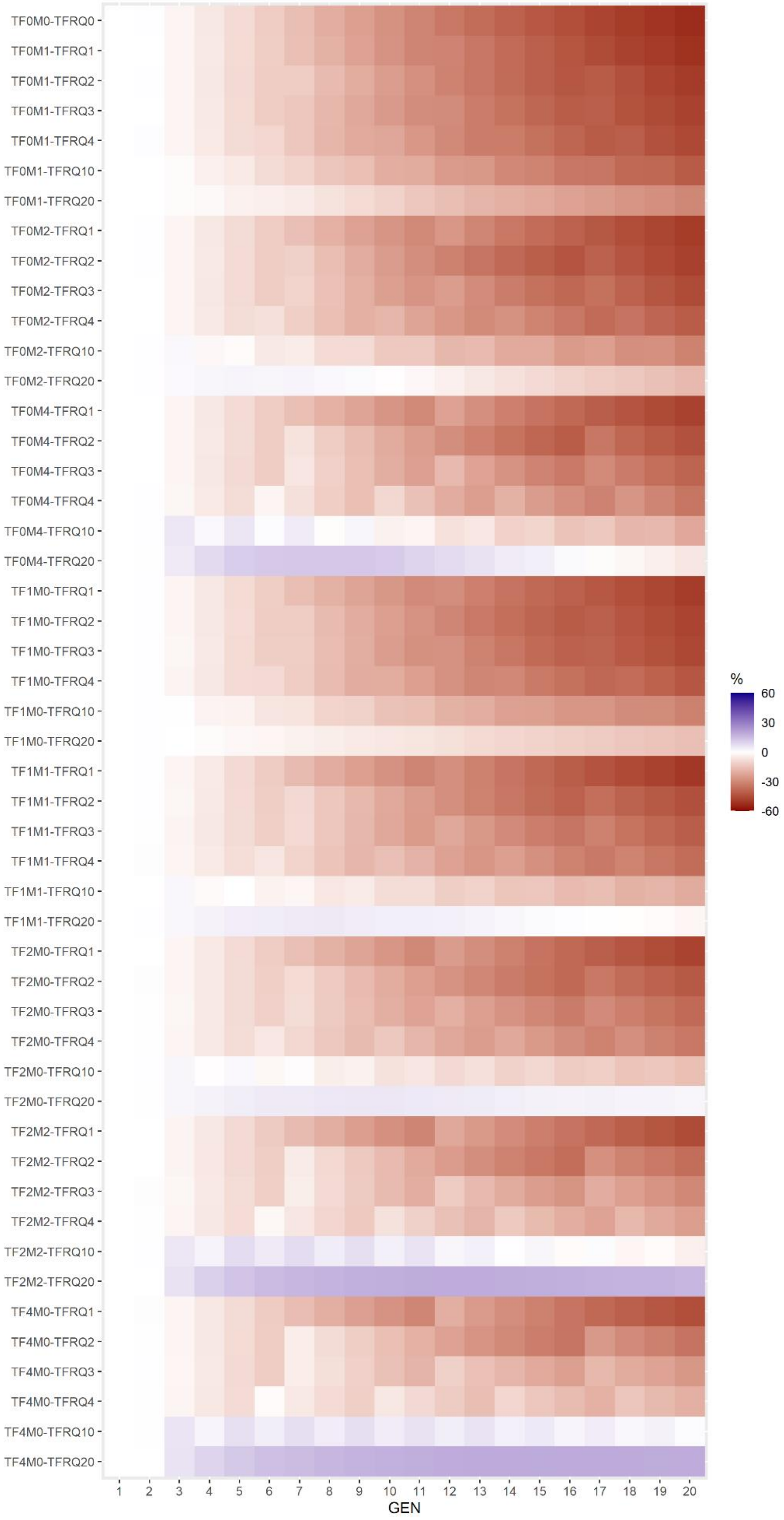

Fig. 16 Percentage change in heterozygosity (*Ho*) relative to generation 1 across number/sex of translocated individuals (TSEX) and frequency of translocations (TFRQ).

References

1. Ash, E., Cushman, S. A., Macdonald, D. W., Redford, T. & Kaszta, Ż. How Important Are Resistance, Dispersal Ability, Population Density and Mortality in Temporally Dynamic Simulations of Population Connectivity? A Case Study of Tigers in Southeast Asia. *Land* **9**, 415 (2020).

2. Ash, E. *et al.* Optimization of spatial scale, but not functional shape, affects the performance of habitat suitability models: A case study of tigers (Panthera tigris) in Thailand. *Landsc. Ecol.* **36**, 455–474 (2021).

3. Reddy, P. A., Cushman, S. A., Srivastava, A., Sarkar, M. S. & Shivaji, S. Tiger abundance and gene flow in Central India are driven by disparate combinations of topography and land cover. *Divers. Distrib.* **23**, 863–874 (2017).

4. Reddy, P. A., Cushman, S. A., Srivastava, A., Sarkar, M. S. & Shivaji, S. Tiger abundance and gene flow in Central India are driven by disparate combinations of topography and land cover. *Divers. Distrib.* **23**, 863–874 (2017).

5. Krishnamurthy, R. *et al.* Multi-scale prediction of landscape resistance for tiger dispersal in central India. *Landsc. Ecol.* **31**, 1355–1368 (2016).

6. SERVIR-Mekong. *SERVIR–Mekong Regional Land Cover Monitoring System (RLCMS)*. https://rlcms-servir.adpc.net/en/landcover/ (2018).

7. OpenStreetMap. OpenStreetMap. www.openstreetmap.org (2019).

8. Ash, E., Cushman, S., Redford, T., Macdonald, D. & Kaszta, Ż. Tigers on the edge: mortality and landscape change dominate individual-based spatially-explicit simulations of a small tiger population. *Landsc. Ecol.* **37**, 3079–3102 (2022).

9. Diniz, M. F., Cushman, S. A., Machado, R. B. & De Marco Júnior, P. Landscape connectivity modeling from the perspective of animal dispersal. *Landsc. Ecol.* **35**, 41–58 (2020).

10. Kramer-Schadt, S., Revilla, E., Wiegand, T. & Breitenmoser, U. Fragmented landscapes, road mortality and patch connectivity: modelling influences on the dispersal of Eurasian lynx. *J. Appl. Ecol.* **41**, 711–723 (2004).

11. Cushman, S. A. Effects of habitat loss and fragmentation on amphibians: A review and prospectus. *Biol. Conserv.* **128**, 231–240 (2006).

12. Kaszta, Ż. *et al.* Integrating Sunda clouded leopard (Neofelis diardi) conservation into development and restoration planning in Sabah (Borneo). *Biol. Conserv.* **235**, 63–76 (2019).

13. Kaszta, Ż. *et al.* Simulating the impact of Belt and Road initiative and other major developments in Myanmar on an ambassador felid, the clouded leopard, Neofelis nebulosa. *Landsc. Ecol.* **35**, 727–746 (2020).

14. Duangchantrasiri, S. *et al.* Dynamics of a low-density tiger population in Southeast Asia in the context of improved law enforcement. *Conserv. Biol.* **30**, 639–648 (2016).
